# Protein disorder in plants: a view from the chloroplast

**DOI:** 10.1186/1471-2229-12-165

**Published:** 2012-09-13

**Authors:** Inmaculada Yruela, Bruno Contreras-Moreira

**Affiliations:** 1 Estación Experimental de Aula Dei, Consejo Superior de Investigaciones Científicas (EEAD-CSIC), Avda. Montañana, 1005, Zaragoza, 50059, Spain; 2 Institute of Biocomputation and Physics of Complex Systems (BIFI), Universidad de Zaragoza, Mariano Esquillor, Edificio I + D, Zaragoza, 50018, Spain; 3 Fundación ARAID, Zaragoza, Spain

**Keywords:** Chloroplast, Intrinsically protein disorder, Plant genome, Gene transfer, Evolution

## Abstract

**Background:**

The intrinsically unstructured state of some proteins, observed in all living organisms, is essential for basic cellular functions. In this field the available information from plants is limited but it has been reached a point where these proteins can be comprehensively classified on the basis of disorder, function and evolution.

**Results:**

Our analysis of plant genomes confirms that nuclear-encoded proteins follow the same trend than other multi-cellular eukaryotes; however, chloroplast- and mitochondria- encoded proteins conserve the patterns of Archaea and Bacteria, in agreement with their phylogenetic origin. Based on current knowledge about gene transference from the chloroplast to the nucleus, we report a strong correlation between the rate of disorder of transferred and nuclear-encoded proteins, even for polypeptides that play functional roles back in the chloroplast. We further investigate this trend by reviewing the set of chloroplast ribosomal proteins, one of the most representative transferred gene clusters, finding that the ribosomal large subunit, assembled from a majority of nuclear-encoded proteins, is clearly more unstructured than the small one, which integrates mostly plastid-encoded proteins.

**Conclusions:**

Our observations suggest that the evolutionary dynamics of the plant nucleus adds disordered segments to genes alike, regardless of their origin, with the notable exception of proteins currently encoded in both genomes, probably due to functional constraints.

## Background

A relevant fraction of genomes encode for proteins with structural disordered regions. Intrinsically protein disorder refers to segments or to whole proteins that do not fold into well-defined regular three-dimensional structures in isolation (*i.e.* not bound to other molecules) [1,2]. This disorder covers local flexible loops, extended domains, molten globule domains and folded domains with flexible linkers [3]. Thus, proteins might be either entirely disordered or partially disordered, characterised by regions spanning just a few (<10) consecutive disordered residues (loops in otherwise well-structured proteins) or long stretches (>30) of contiguously disordered residues. The presence of protein disorder is thought to confer dynamic flexibility to proteins, allowing transitions between different structural states [4]. This increased flexibility is advantageous to proteins that recognise multiple target molecules such as DNA, RNA, other proteins or small ligands [3,5]. It is predicted that between 30% and 60% of proteins contain stretches of 30 or more disordered residues, with multi-cellular eukaryotes having much more predicted disorder than unicellular eukaryotes [6]. There is evidence that the unstructured state, common to all living organisms, is essential for basic cellular functions [5,7]. Whole-cell NMR experiments demonstrate that intrinsic disorder can exist *in vivo*[3,8] and therefore this state does not result merely from the failure to find the correct conditions for folding or ligand binding. Despite their lack of a well-defined three dimensional (3D) structure, these proteins carry out basic functions, mostly associated with regulatory processes in the cell, including transcription, translation, cellular signal transduction, protein phosphorylation, the storage of small molecules, and the regulation of the self-assembly of large multi-protein complexes such as the ribosome, in which interactions with multiple partners and high-specificity / low-affinity interactions are often required. The functional diversity provided by disordered regions complements that of ordered protein regions [9-11]. It has been also reported the importance of disordered interfaces in the modulation of cellular regulatory response, which participate in subtle regulation by switching its specificity for different binding partners [12].

In plants, the available information about intrinsic disorder in proteins is rather limited compared to other eukaryotic organisms and concerns basically to *Arabidopsis thaliana*, which was the first complete genome sequenced. Particularly, it has been pointed out that late embryogenesis abundant (LEA) proteins, with chaperone activity, and dehydrin proteins, lack a stable three-dimensional structure being probably fully disordered [13-15]. These proteins are associated with abiotic stress tolerance, particularly with cold stress and dehydration. The computational prediction of disorder by Dunker *et al.*[1] did not reveal notable disorder differences among the proteome of *A. thaliana* and those of other eukaryotes. However, currently it is not known whether this scenario is general for all plant proteomes. Additionally, another overlooked aspect is the comparison of the degree of disorder in organelle and nuclear proteomes. Evolutionary analysis of *A. thaliana,* cyanobacterial and chloroplast genomes have revealed that many genes were transferred from plastids to the nucleus during plant evolution [16]. In particular, it has been estimated that in *A. thaliana* approximately 18% of the total protein-coding genes were acquired from the cyanobacterial ancestor of plastids.

At present computational analysis are considered crucial and indispensable for the identification and characterization of unstructured proteins [2,17]. Several methods have been developed to predict intrinsic disorder from amino acid sequences, such as DisEMBL [18]; GLOBPROT2 [19]; DISOPRED2 [20,21]; IUPred [22]; PONDR VL-XT [23-25], among others. Among these we decided to use the DISOPRED2 software, which has achieved specificities of 0.95 at the residue level in four successive Critical Assessment of Techniques for Protein Structure Prediction experiments (CASP6-9), and has been shown to be the best predictor of long disordered regions in CASP9 [26,27].

 Here we report the disorder analysis of proteins from 8 vascular plants, 1 bryophyta and 3 chlorophyta encoded in either plastid, mitochondrial or nuclear genomes by using the DISOPRED2 method. In order to gain biological and evolutionary insights, we focus on the subset of chloroplast genes which moved to the nucleus during plant evolution. It is observed that originally chloroplast-encoded proteins acquired disorder after their genes moved to the nucleus. In contrast, proteins still encoded in the chloroplast chromosome barely become disordered. Finally, in order to further evaluate these findings, we review the incorporation of disorder to chloroplast ribosomal subunits, one of the most representative transferred gene clusters, in comparison to their bacterial counterparts.

## Results

### Analysis of disorder and occurrence of amino acids in protein sequences

**Figure 1:**
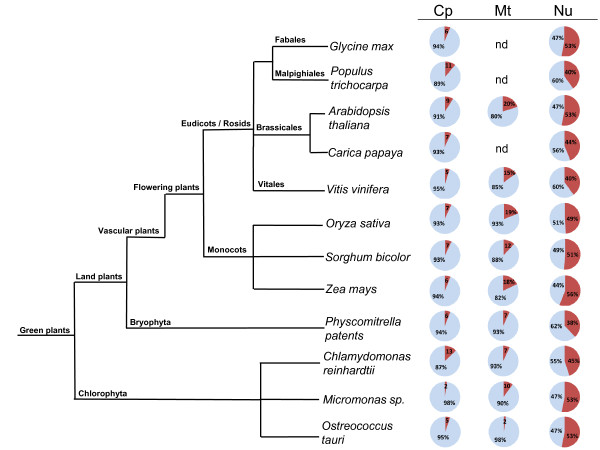
**Distribution of predicted disordered segments with L ≥ 30 in plants.** Disorder in chloroplast (Cp), mitochondria (Mt) and nuclear (Nu) proteomes are shown. Percentages of intrinsically disordered proteins are in red and percentages of non-disordered proteins are in light blue.

We have analyzed the occurrence of protein disorder in 12 complete plant proteomes (see Materials and Methods). Chloroplast (*ca*. 85 proteins in average), mitochondrial (*ca.* 64 proteins in average) and nuclear (*ca.* 25,000 proteins in average) proteomes were separately analyzed and the occurrence of disordered regions of different length (L) was calculated. In plant nuclear proteomes the percentages of predicted disordered segments with L ≥ 30, L ≥ 40, and L ≥ 50 were determined (full detail in Additional file 1: Table S1). The data showed in average a range of disorder ranging from 40 to 56%, 26 to 44% and 19 to 33%, respectively. Figure 1 summarizes the data corresponding to predicted to-be-disordered segments with L ≥ 30. The highest percentages of disorder were found in *Zea mays* (56.2%), *Glycine max* (53.3%), *Physcomitrella thaliana* (52.6%), *Micromonas* sp. RCC299 (52.9%) and *Ostreococcus tauri* (52.5%). In general, no statistically significant differences between vascular plants (8) and bryophyta (1) and chlorophyta (3) species were found (Χ^2^ values of 2.367 for bryophyta and 0.060 for chlorophyta, see Additional file 2: Table S2). Nonetheless *Physcomitrella patens* had the lowest percentage, 38.2%, a value close to those found in Archaea and bacteria. It is also worth mentioning that no obvious differences were observed between monocots and eudicots.

Chloroplast (2 - 13%) and mitochondrial (2 - 19%) proteomes clearly exhibit much less disorder than nuclear ones (Additional file 1: Table S1). In chloroplasts for L ≥ 30, *Micromonas* sp displays the lowest amount of disorder (2%) and perhaps surprisingly *Vitis vinifera* showed values (4.6%) close to those found in microalgae. Concerning mitochondria, the lowest percentage (2.3%) was found in *Ostreococcus tauri*.

In an attempt to validate our disorder predictions, we searched in the Protein Data Bank (PDB) for homologous proteins to those of *A. thaliana* identified as intrinsically disordered proteins in our analysis, as explained in Materials and Methods. This was a very limited validation effort, since it was only possible to recover data for 70 sequences. Nevertheless, we found that 49/70 (61/70 if we consider terminal sequences partially aligned to predicted disordered regions) contained segments with unresolved 3D-structure.

The distribution of disordered segments of L ≥ 30 along complete protein sequences was calculated, splitting proteins in *N-*terminal (40 aa), *C-*terminal (40 aa) and internal regions. The results in Table 1 indicate that in nuclear proteomes the disordered regions are slightly more abundant in the internal regions of proteins (50 - 65%) compared with the extremes of the protein sequence (14 - 30%), being the *N*-terminal part (20 - 31%) more disordered than the *C*-terminal one (14 - 20%). This distribution differs to that calculated for chloroplasts and mitochondria; in organelles the results indicate a more similar occurrence of disorder in the internal regions (21 - 41% in chloroplasts, and 28 - 46% in mitochondria) compared with the terminal regions (15 - 44% in chloroplasts and 24 - 41% in mitochondria). This scenario was common for all the plant proteomes studied with the exception of the chloroplast from *C. reindhartii*, where the disorder distribution was similar to that observed in the nuclear proteome (*i.e.,* the internal part was more disordered than the terminal regions).

**Table 1:** Distribution of disordered segments with L ≥ 30 in protein sequences from plant proteomes

**Proteomes**	**AT**	**CP**	**PT**	**VV**	**OS**	**SB**	**ZM**	**GM**	**PP**	**CR**	**MCR**	**OT**
**Nuclear proteomes**												
N-terminal (40 aa)	25512 / 97853 26.07%	24444 / 80869 30.22%	34479 / 126754 27.20%	24524 / 92151 26.61%	42303 / 148406 28.50%	29680 / 115497 25.70%	29628 / 114125 25.96%	40313 / 167551 24.06%	31926 / 130425 24.48%	13158 / 63766 20.63%	9566 / 46371 20.63%	8069 / 33395 24.16%
Internal part	56422 / 97853 57.66%	40528 / 80869 50.11%	68242 / 126754 53.84%	51256 / 92151 55.62%	79257 / 148406 53.40%	66199 / 115497 57.32%	65801 / 114125 57.66%	100443 / 167551 59.95%	76575 / 130425 58.71%	41269 / 63766 64.72´%	30027 / 46371 64.75%	19871 / 33395 59.50%
C-terminal (40 aa)	15919 / 97853 16.27%	15897 / 80869 19.66%	24033 / 126754 18.96%	16371 / 92151 17.76%	26846/ 148406 18.09%	19618 / 115497 16.98%	18696 / 114125 16.38%	26795 / 167551 15.99%	21924 / 130425 16.81%	9339 / 63766 14.64%	6778 / 46371 14.62%	5455 / 33395 16.33%
**Chloroplast proteomes**												
N-terminal (40 aa)	46 / 138 33.33%	50 / 154 32.47%	66 / 170 38.82%	49 / 156 31.41%	66 / 151 43.71%	49 / 118 41.52%	64 / 148 43.24%	48 / 141 34.04%	52 / 145 35.86%	36 / 205 17.56%	29 / 76 38.15%	33 / 96 34.37%
Internal part	55 / 138 39.85%	63 / 154 40.91%	55 / 170 32.35%	63 / 156 40.38%	33 / 151 21.85%	28 / 118 23.73%	38 / 148 25.67%	56 / 141 39.72%	51 / 145 35.17%	137 / 205 66.83%	20 / 76 26.31%	33 / 96 34.37%
C-terminal (40 aa)	37 / 138 26.81%	41 / 154 26.62%	49 / 170 28.82%	44 / 156 28.20%	52 / 151 34.43%	41 / 118 34.74%	46 / 148 31.08%	37 / 141 26.24%	42 / 145 28.96%	32 / 205 15.61%	27 / 76 35.52%	30 / 96 31.25%
**Mitochondrial proteomes**												
N-terminal (40 aa)	85 / 236 36.02%	-	-	49 / 143 34.26%	37 / 129 28.68%	22 / 74 29.73%	147 / 364 40.38%	-	25 / 71 35.21%	5 / 20 25.00%	21 / 62 33.87%	18 / 50 36.00%
Internal part	78 / 236 33.05%	-	-	46 / 143 32.17%	58 / 129 44.96%	34 / 74 45.94%	124 / 364 34.06%	-	25 / 71 35.21%	7 / 20 35.00%	22 / 62 35.48%	14 / 50 28.00%
C-terminal (40 aa)	73 / 236 30.93%	-	-	48 / 143 33.57%	34 / 129 26.35%	18 / 74 24.32%	93 / 364 25.55%	-	21 / 71 29.58%	8 / 20 40.00%	19 / 62 30.64%	18 / 50 36.00%

Amino acid frequencies in disordered proteins were also analyzed. The amino acid residues Ser, Pro, Gln, Lys and Glu are over-represented in intrinsically disordered regions from nuclear proteomes. In contrast, the amino acid residues with lowest frequencies were Trp, Cys, Tyr, Phe, Ile, Leu and Val (Additional file 3: Figure S1A). In chloroplasts and mitochondria some differences were observed: Lys and Met showed higher frequencies, being Ser and Pro less abundant (Additional file 3: Figures S1B and S1C).

### Disorder in proteins encoded by plastidic genes in the nucleus

**Figure 2:**
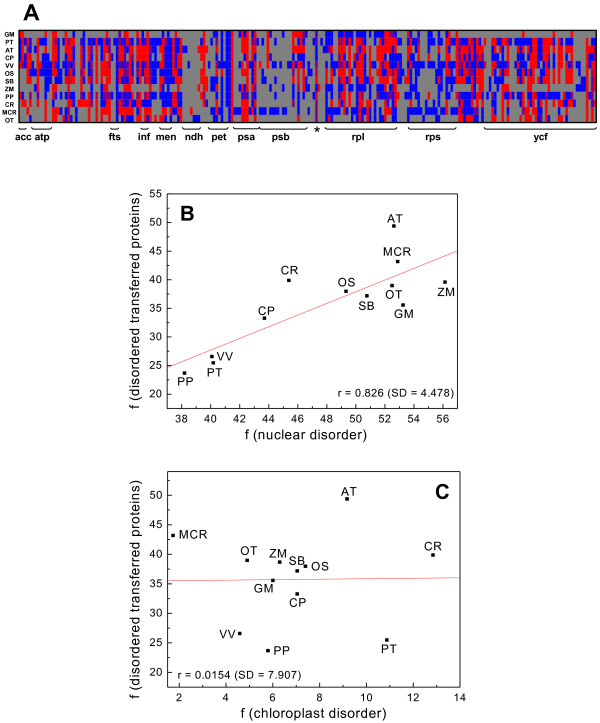
**Disorder frequencies in chloroplast and nuclear proteomes.** A) Heatmap of 226 proteins transferred from plastid to nucleus (X- axis) in 12 analysed plant genomes (Y-axis). Colour scale goes from blue (order) to red (disorder); magenta is shown in cases were not all members within a group of paralogous genes encode disordered proteins. Absent orthologues are painted in grey. RUBISCO small subunit is marked with an asterisk. B) Scatter plot of disorder frequencies in complete plant nuclear proteomes (X-axis) *versus* disorder frequencies in the nuclear proteome fraction with chloroplast origin (Y-axis). C) Linear plot of disorder frequencies in complete plant chloroplast proteomes (X-axis) *versus* disorder frequencies in the nuclear proteome fraction with chloroplast origin (Y-axis). A protein is considered disordered if it contains a contiguous stretch of predicted disordered residues of L ≥ 30 amino acids.

Intrinsic disorder was investigated in proteins believed to be originally encoded in chloroplast genomes, which were subsequently transferred to the nuclear genome in the course of evolution. With this aim we retrieved from the PLAZA database (for details see Materials and Methods) all *Arabidopsis thaliana* protein-coding genes within the nuclear genome with a plastid origin as reported in Martin *et al.*[16]. The analysis revealed that in *A. thaliana* 147 of 298 total proteins (49.3%) contain L ≥ 30 segments disordered. The analysis for the rest of plant proteomes was done with the transferred nuclear genes identified by homology (see Materials and methods). We found that disordered proteins were 84 of 253 (33.2%) in *Carica papaya,* 72 of 203 (35.5%) in *Glycine max*, 122 of 480 (25.4%) in *Populus trichocarpa,* 107 of 404 (26.5%) in *Vitis vinifera*, 118 of 311 (37.9%) in *Oryza sativa*, 106 of 286 (37.1%) in *Sorghum bicolor*, 78 of 202 (38.6%) in *Zea* mays, 112 of 379 (23.6%) in *Physcomitrella patens*, 76 of 191 (39.8%) in *Chlamydomonas reindhartii*, 62 of 144 (43.1%) in *Micromonas* sp. RCC299, 56 of 150 (38.9%) in *Ostreococcus tauri*. The lowest disorder was calculated for *Physcomitrella patens* (23.6%) and the highest for *Arabidopsis thaliana* (49.3%)*.* As illustrated in Figure 2A, the acquisition of disorder by transferred proteins is not uniform across plant species. In 125 out of 226 orthologous groups of transferred genes there are instances where a protein contains long disordered segment in some species but not in others. 

The percentages of disorder in transferred proteins seem to follow the same trend observed for overall disorder in the corresponding proteomes. In order to further validate this observation we plotted the disorder frequencies of nuclear proteins for L ≥ 30 *versus* the frequencies of disorder in proteins originally encoded by chloroplast genes and currently placed in nuclear genomes (Figure 2B). The Pearson correlation obtained was r = 0.826. However, when we plotted the frequencies of protein disorder in the chloroplast for L ≥ 30 *versus* the disorder frequencies of transferred chloroplast genes (Figure 2C), the obtained correlation coefficient was insignificant (r = 0.0154).

**Figure 3:**
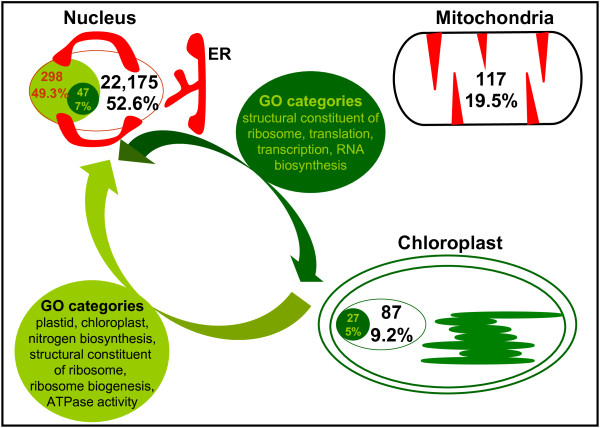
**Scheme of intrinsically disordered proteins and disorder transfer from chloroplast to nucleus in* Arabidopsis thaliana. *** Total proteins encoded in nucleus, chloroplast and mitochondria and percentages of disorder (L ≥ 30) are written in black. The number of proteins transferred from chloroplast to nucleus and the respective percentage of disorder are written in red (light green arrow). The number of those nucleus-encoded proteins with a putative orthologous copy in the chloroplast and the respective percentage of disorder are written in green (dark green arrow). The most predominant Gene Ontology categories of proteins transferred to nucleus are annotated.

Martin *et al*. [16] reported that some genes encoding for cyanobacterial proteins and identified in the plant nuclear genome still conserve a copy in the chloroplast genome. We have found that this group of proteins has a much lower percentage of disorder (*ca.* 7%) than those that have lost their original chloroplast sequences (20 - 52%). In the case of *A. thaliana* our results revealed that a group of 47 nuclear-encoded proteins maintain putative orthologous copies in the chromosome of the chloroplast. In particular we found that these nuclear proteins correspond to 27 chloroplastic non-disordered proteins, indicating that some of them might be paralogues. For instance, this is the case of the chloroplast NAD(P)H-quinone oxidoreductase subunit 2B (AtCg01250), the NAD(P)H dehydrogenase (AtCg01090), the RNA polymerase beta’ subunit (AtCg00180) or the second-largest subunit of DNA-dependent RNA polymerase (AtCg00190). In addition, ribosomal proteins L14 (AtCg00780), L22 (AtCg00810), S8 (AtCg00770) and S19 (AtCg00820), which are among the most conserved ribosomal proteins and bind directly to 23S and 16S rRNAs, respectively, are included in this group [28-30] (Additional file 4: Table S3). As mentioned above, these conserved proteins barely acquire disorder. The scheme in Figure 3 summarizes the protein transfer scenario from chloroplast to nucleus in *A*. *thaliana.*

**Figure 4:**
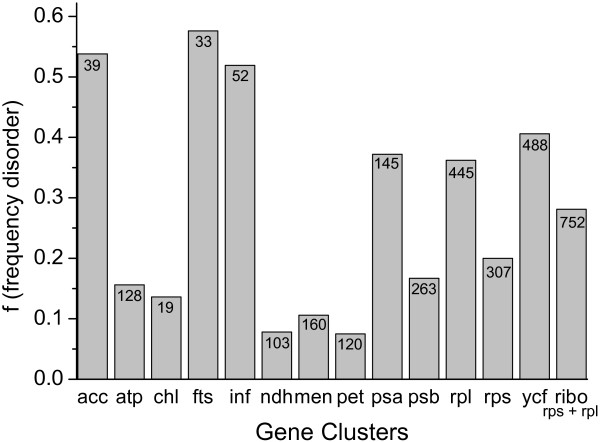
**Distribution of disordered proteins in the fraction of the nuclear genome with plastidic origin, in terms of gene clusters.** The number of proteins grouped in each gene cluster is given in each bar.

We have further grouped transferred intrinsically disordered proteins in gene clusters (Figure 4), reminiscent of the ancestral bacterial operons, finding that the *fts*, *inf, acc, psa, rpl and ycf* gene clusters encode more frequently for disordered proteins (40 - 58% of disorder). These genes are involved in cell division, translational initiation and acetyl-CoA carboxylase pathways, or photosystem I, large ribosomal subunits. In contrast, the *atp, chl, ndh*, *men, pet, psb and rps* gene clusters, which encode for ATP synthase subunits, protochlorophyllide reductase, NADH-plastoquinone oxidoreductase subunits, succinyl or naphtoate synthase, cytochrome *b*_6_/*f*, photosystem II subunits and ribosomal small proteins, contain less disordered proteins (8 - 25% of disorder). These observed differences do not appear to be related to protein length, as the average length of intrinsically disordered proteins was found to be 390 aa, a similar value to that of non-disordered proteins (391 aa).

### Gene ontology annotations of disordered proteins of plastidic origin

In order to put in perspective the previous observations we investigated the annotated function of disordered proteins in the 12 plant species studied by using the Gene Ontology (GO). In the course of this examination a protein was considered disordered if it contained a contiguous stretch of predicted disordered residues of L ≥ 30 amino acids. The analysis revealed that disordered proteins encoded in nuclear genes assumed to be of plastidic origin were enriched in 29 biological processes (P), 39 cellular components (C) and 13 molecular functions (F) GO categories with corrected *p*-values < 10E-5 (see Additional file 5: Table S4). As to the cellular component, we found that these proteins were mainly associated to “plastid” (4.60E-43) and “chloroplast” classes, which supports our homology-based selection of chloroplast-transferred genes. The most significant association among specific biological processes was with “cellular nitrogen compound biosynthetic process” (1.10E-13), including cofactor, heterocycle and tetrapyrrole biosynthetic processes. Finally, a few molecular functions were found to be associated to these disordered proteins, such as “structural constituent of ribosome” (8.01E-09) and “ATPase activity” (4.35E-06). These reported corrected p-values are relative to *A. thaliana*, which is probably the best-annotated plant genome for its role as a model organism. Altogether, these results suggest that disordered transferred proteins as a whole are not strongly linked to any one function. Moreover, nuclear-encoded genes still maintaining a copy in the plastid chromosome were mainly associated to GO cellular components “ribosome” (5.43E-30) and “ribonucleoprotein complex” (2.24E-26). Among biological processes, they were mainly associated to “gene expression” (5.35e-36) including “translation” (2.61E-25), “transcription” (5.97E-14) or “RNA biosynthesis” (9.8E-11). Finally, at the level of molecular function, these proteins were found to be annotated as “structural constituent of ribosome” (2.95E-32), “structural molecule activity” (1.56E-28), “DNA-directed RNA polymerase activity” (2.18E-15) or “NADH dehydrogenase activity” (2.45E-7) (Figure 3).

We also reviewed the annotated function of non-disordered proteins of chloroplast origin and the results were more compelling, as this set of proteins is more homogeneous (see Additional file 6: Table S5). Among biological processes, several translation-related annotations were considerably associated, such as “ribosome biogenesis” (1.28E-31). These agree well with the most significant cellular component found, which “cytosolic large ribosomal subunit” is (1.05E-46). In addition, the strongest association found at the level of molecular function was “structural constituent of ribosome” (4.59E-45).

Additionally, the functions of intrinsically disordered nuclear-encoded proteins were also analyzed (data not shown). Among biological processes the most notable annotations were related to “regulation”, including “regulation of nucleobase” (1.96E-267), “regulation of nitrogen compound” (2.48E-266), “regulation of macromolecule biosynthetic process” (5.94E-265) or “regulation of RNA metabolic process” (9.61E-265). At the level of cellular component, significant associations were found with “nucleus” (7.63E-162), “membrane-bound organelle” (5.78E-144) and “organelle” (8.79E-129). These annotations correspond well with those of molecular function categories, such as “nucleic acid binding transcription factor activity” (1.19E-260), “nucleic acid binding” (1.38E-250) or “DNA binding” (2.23E-209). Overall, these functional classes match those reported for eukaryotes in general [5].

### Disorder in ribosomal proteins

An in-depth analysis of chloroplast ribosomal proteins was performed with the aim of better understanding the evolution of protein disorder in plants. These proteins were selected for three reasons: *i)* they are the largest gene cluster transferred to the nuclear genom*e; ii)* they are part of a highly conserved and essential cellular system, and *iii)* they were highlighted in the GO annotation study described above. The idea was to compare *A. thaliana* (eudicot) and *O. sativa* (monocot) proteins with their orthologues in prokaryotic ribosomes (4 Archaea, 3 Gram +, 4 cyanobacteria, 7 eubacteria and 4 proteobacteria). For details see Materials and Methods and Additional file 7: Tables S6A and S6B. We have calculated that 30% and 65% of these proteins are intrinsically disordered in chloroplast 30S and 50S subunits, respectively. The data show that protein disorder is not uniform across bacteria species. There are instances where a protein contains long disordered segment in some species but not in others. It is worth mentioning that no differences were found between the two plant species.

**Figure 5:**
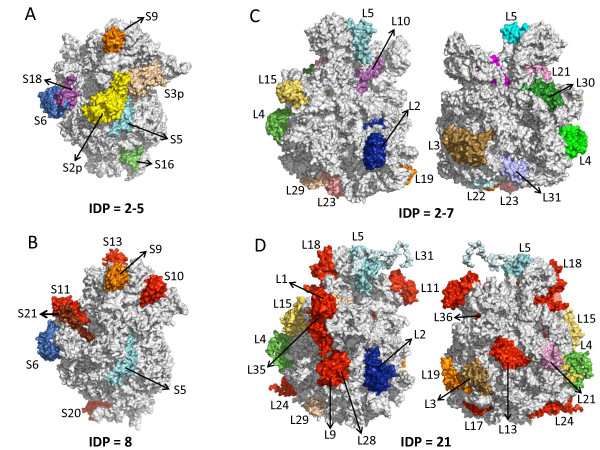
**Distribution of disordered proteins on the bacterial (A,C) and chloroplast (B,D) ribosome (mapped over PDB entries 1JOO, 1VQ8, 3BBN and 3BBO, respectively).** Panels A and B correspond to the 30S subunit, C and D to the 50S. Disordered proteins in bacterial ribosomal subunits are highlighted in pale yellow, pale blue, light blue, dark blue, orange, green, magenta and pink. Their chloroplast orthologues in *Arabidopsis thaliana* are marked in the same colour. Additional proteins found to be intrinsically disordered in chloroplast 30S and 50S subunits are highlighted in red. Numbers following S and L identify small and large subunit proteins, respectively. The disordered protein L7/L12 in the chloroplast 50S subunit is not marked because of it is absent in the structural data retrieved from the PDB. The average number of intrinsically disordered proteins (IDP) calculated for each ribosomal subunit is written below (for details see in Additional file 7: Table S6). The three-dimensional cartoons were drawn using PyMol 1.4.1 (Schrodinger LLC).

Figures 5A/C and 5B/D colour ribosomal proteins that were predicted to be disordered in our analysis, (and observed experimentally in some cases as described in [3], in at least one prokaryote (top) and one plant chloroplast (bottom) genome, respectively. It can be observed that the disorder degree of the small (30S) subunit does not increase in chloroplast ribosomes (Figure 5B). On the contrary, the disorder increases notably in the chloroplast large (50S) subunit (Figure 5D). An interesting feature that might explain this finding is that the majority of L-proteins are nuclear encoded (33/42) being this ratio lower (12/22) in the case of S-proteins. Interestingly, in certain plant genomes (*i.e., O. sativa, S. bicolor, Z. mays, P. trichocarpa, V. vinifera, G. max, P. patens*) it was found that some ribosomal proteins are encoded by both nuclear and plastid genes, and in the majority of cases the resulting protein products are identical. 

In the small subunit, we found that chloroplast proteins S10, S11, S13 and S20 have acquired disorder with respect to their prokaryotic orthologues, but have also lost disordered segments observed in bacteria (for instance in S2, S3 and S18). Note that plant S10, S13 and S20 protein sequences are much longer than their prokaryotic counterparts (see in Additional file 8: Table S7), and this might explain the gain of disordered segments. Overall, there is not a clear net gain of disorder in this subunit (see in Additional file 7: Table S6A). Within the large subunit, L1, L6, L7/L12p, L9, L11, L13, L17, L18, L24, L27, L28, L34, L35 and L36 proteins gain disorder in the chloroplast. With the exception of L36, all these are nuclear-encoded.

## Discussion

The analysis of 12 plant proteomes reveals a similar occurrence of disordered proteins to that found in other eukaryotic organisms [1]. Therefore, there is no clear separation among animals, yeast and plants in terms of the total amount of predicted disordered segments. Nor clear differences were observed among different plant species belonging to bryophyta, chlorophyta and vascular plant, or among eudicots and monocots.

The amino acid composition of disordered segments in plants corresponds well with that reported for other eukaryotes [3,5,11], which can be defined by a low frequency of bulky hydrophobic residues, which normally form the core of a folded protein, and high frequency of polar residues contributing to net charge. The minor presence of cysteine residues within disordered regions was also a characteristic feature observed in either chloroplast, mitochondrial or nuclear proteins, which fits well with other predicted disordered protein profiles [5]. This finding supports that these features in disordered protein regions are stable during evolution. On the other hand, the distribution of disordered regions along the complete protein sequence was slightly higher in the internal parts than in the terminal parts of proteins. This feature was common for all the plant proteomes investigated and no differences were found among different species. This observation differs from the data obtained from protein 3D structures from the Protein Data Bank [31]. These authors reported that the fraction of disordered residues is more abundant in the terminal parts (72%), constituted by 40 residues near to the *N*-terminal and the *C*-terminal compared with the middle part (all other residues).

Interestingly, a survey of chloroplasts and mitochondria revealed significant differences concerning the occurrence of disordered regions when compared with the nuclear genome. The percentages calculated in these organelles are in the order of magnitude of those determined in Archaea and bacteria [1]. These data are in agreement with the bacterial origin of genes coding for these proteins. We also observed differences concerning the distribution of disordered regions in the protein chain.

It has been suggested that between 800 and 2,000 genes in the *Arabidopsis thaliana* genome might come from cyanobacteria, with a majority of proteins included in the functional category of biosynthesis and metabolism [32-35]. Furthermore, the analysis of 15 sequenced chloroplast genomes revealed 117 nuclear-encoded proteins that are also still present in at least one chloroplast genome [16]. Based on these reports we evaluated the degree of disorder in both nuclear-encoded proteins, which were transferred from the plastid to the nuclear genome, and those transferred to the nucleus that also still conserve a copy in the chloroplast genome. Our results indicate that transferred proteins acquired disorder with a frequency similar to that of nucleus-encoded proteins. During evolution, organelles export their genes to the nucleus, but many of these proteins are imported to the chloroplast, with the help of transient peptides and protein-import machinery, to carry out their function. This gain of disorder can be hypothesized to be an advantage during the import-pathway across a double-membrane barrier. However, these disordered segments are not preferentially associated to transient peptides localized in the N-terminal region. Indeed, they were found to be slightly more abundant in the internal region of the protein chain. Moreover, those transferred protein coding-genes that maintain a copy in the chloroplast genome exhibit much lower disorder than those that have lost the plastid copy, similar to proteins encoded by chloroplast or bacterial genes. This fact might be revealing a selection pressure during evolution. These proteins are mainly involved in translation, transcription or RNA biosynthesis, being structural constituents of the ribosome and the ribonucleoprotein complex. The disorder in proteins encoded by ancient chloroplast genes but currently in the nucleus follows the order bryophyta < vascular plants < chlorophyta. In this context, the data suggest that the level of disorder introduced into plastid proteins that have moved to the nuclear genome has increased during evolutionary time, but further investigations will be necessary to clarify this issue.

The gain or loss of disorder in transferred proteins might be to some extent a stochastic process, since orthologous copies found in different plant species do not necessarily conserve disordered segments, despite presumably carrying out similar functions. This observation is in agreement with the finding that gene transfer events from the chloroplast to the nuclear genome occur much more frequently than generally believed, contributing significantly to genetic variations [35]. In this respect it is also noted that disorder distribution in ribosomal proteins among bacterial species appears rather at random (Additional file 7: Table S6).

Non-folding unstructured proteins and regions might be expected to change more rapidly during evolution than structured proteins because buried amino acid residues are highly constrained while disordered regions are not constrained by the structure [11]. It is believed that disordered proteins do not exist as a single structure but rather as a conformational equilibrium of states, which interconvert into each other over a range of time scales. This feature can be an evolutionary advantage for adaptation, for instances, under stress conditions. Additionally, intrinsically disordered proteins could be more susceptible to proteolytic degradation *in vitro.* The classical PEST hypothesis states that the presence of segments rich in Pro, Glu(Asp) and Ser/Thr flanked by Arg/Lys residues in proteins correlates with a short lifetime in the cell [36,37]. Accordingly, the fact that a group of proteins related to the ribosome biogenesis preserved its ordered character when transferred to the nucleus could be explained by this critical role within the protein synthesis machinery which should be maintained.

On the other hand, around 25% of chloroplast ribosomal proteins transferred to the nucleus are predicted to be intrinsically disordered in our analysis. In this respect it has been argued that flexibility favours the structural assembly of components of large complexes such as those involved in ribosome and therefore such characteristic should be prevalent in certain ribosomal proteins [38]. Moreover, RNA-binding proteins usually contain unstructured regions as is the case of the ribosomal protein L5, which is reported to be associated with 5S rRNA [39]. Our results also indicate that intrinsic disorder is a well-conserved character in some ribosomal proteins. This is the case of L4 and L15, predicted to contain unstructured segments in all the bacterial and plant proteomes analysed. Ribosomal protein L4 is localized near the peptidyl transferase center of the bacterial ribosome [40] and displays significant RNA chaperone activity [41]. The L15 protein is involved at later stages during assembly [41].

The comparison of disorder between bacterial and chloroplast ribosomal proteins unveiled a disorder increase in the chloroplast large 50S subunit, where proteins are in average 55 residues longer, as previously reported by Yamaguchi and Subramanian [42], and the majority are produced by nuclear genes. This finding contrasts with the data obtained with the whole proteome, which show no differences in length between disordered and non-disordered proteins. In the case of the small 30S subunit such differences were not so clear, probably due to the higher content of chloroplast-encoded proteins, which most of them are predicted to be non-disordered. These results support our hypothesis that proteins encoded in the nuclear genome are more likely to stochastically acquire disorder. On the other hand, however, we cannot preclude that differences in rRNA composition between chloroplast (23S, 5S and 4.5S) and bacterial (23S and 5S) large 50S ribosomal subunit could also explain the gain of disorder observed in this subunit [43,44].

Differences in the genetic machinery between plastids (prokaryotic) and nucleus (eukaryotic) could also help to explain our observations. When plastid genes reach the nucleus they move from a genetic apparatus that is compact, operon-harbouring and intron-poor, to one that is more complex, operon-splitting and intron-rich [45]. While the gain of disorder is thought to be advantageous or neutral in many cases, there must be selective pressures that put restrictions to this apparently random process, as is the case of the chloroplast RUBISCO small subunit protein, a nuclear-encoded protein with a plastid origin, which was found to be ordered in most of the plant proteomes investigated (see Figure 2).

The comparison of 3D structures of bacterial and chloroplast ribosomal subunits revealed the localization of the extra disordered proteins. For instance, S11 is localized in the mRNA path, next to the intrinsically disordered S21, which directly interacts with the 5’ untranslated region of the mRNA [46]. In the ribosomal 50S subunit, L24 and L29 are localized surrounding the polypeptide tunnel exit site. It is worth noting that some of these chloroplastic disordered proteins are normally found in cyanobacteria (see in Additional file 7: Table S6), but in some cases are unstructured in gram-positive bacteria and not in cyanobacteria (*i.e.* S9, L29 and L31). This might be related with the fact that more *Arabidopsis* proteins branched with their homologues from gram-positive bacteria (*Mycobacterium*) than did with cyanobacteria (*Prochlorococcus, Synechocystis*)*.* This has been interpreted as if the *Arabidopsis* lineage acquired genes specifically from gram-positive bacteria subsequent to its divergence from the yeast lineage [16]*.*

## Conclusions

Taken together, our chloroplast-based analyses demonstrate that disordered segments are acquired by proteins most probably due to the process of nuclear integration during plant evolution. However, we observed that some parts of the ancestral chloroplast and mitochondria organelles present in eukayotic cells are being preserved from acquiring disordered segments, probably due to functional constraints and evolutionary pressure.

## Methods

### Proteomic and GO databases

Chloroplast, mitochondrial and nuclear complete plant proteomes, and the Gene Ontology (GO) annotations for *Arabidopsis thaliana* (AT)*, Carica papaya* (CP)*, Chlamydomonas reindhartii* (CR)*, Oryza sativa* (OS)*, Populus trichocarpa* (PT)*, Physcomitrella patens* (PP)*, Sorghum bicolor* (SB)*, Vitis vinifera* (VV) were retrieved from PLAZA v.1, and *Glycine max* (GM)*, Micromonas* sp. RCC299 (MRC)*, Ostreococcus tauri* (OT) *and Zea mays* (ZM) from PLAZA v.2 (http://bioinformatics.psb.ugent.be/plaza/).

### Gene transfer analysis

Based on the data reported in Martin *et al.*[16] the protein-coding genes in sequenced chloroplast genomes and identified nuclear homologues in *A. thaliana* (AT) were retrieved using the tools available in (http://bioinformatics.psb.ugent.be/plaza/). The corresponding homologues were identified in *C. papaya* (CP)*, C. reindhartii* (CR)*, O. sativa* (OS)*, P. trichocarpa* (PT)*, P. patens* (PP)*, S. bicolor* (SB)*, V. vinifera* (VV), *G. max.* (GM)*, Micromonas* sp. RCC299 (MRC)*, O. tauri* (OT) *and Z. mays* (ZM) and retrieved from PLAZA. To identify those proteins encoded by nuclear genes, which still maintain a homologous copy in the chloroplast genome, we used BLAST bidirectional best hits, taking either the chloroplast protein or the nuclear protein as query.

Ribosomal protein sequences from bacteria *Pyrococcus furiosus* (Pyf), *Methanobacterium sp*. (Meb), *Methanocaldococcus jannaschii* (Mtj); *Archaeoglobus fulgidus* (Af), *Mycoplasma pneumoniae* (Myc), *Bacillus subtilis* (Bas), *Mycobacterium tuberculosis* (Myt), *Nostoc punctiforme* (Nos), *Prochlorococcus marinus* (Pro), *Synechocysistis sp*. PCC 6803 (Syn); *Synechococcus sp*. (Sych), *Borrelia burgdorferi* (Bob), *Chloroflexus aggregans* (Chla), *Chlorobium chlorochromatii* (Chlb); *Treponema pallidum* (Trep), *Chlamydia pneumoniae* (Chlp), *Clostridium hathewayi* (Clos); *Aquifex aeolicus* (Aqa), *Rickettsia prowazekii* (Rip), *Heliobacter pylori* (Hep), *Haemophilus influenzae* (Hai), *Escherichia coli* (Ec) were retrieved from NCBI (http://www.ncbi.nlm.nih.gov). This set of prokaryotes is chosen for analysis in the work of Martin *et al.* (2002). The corresponding homologues in *A. thaliana* and *O. sativa* were retrieved using the tools available in (http://bioinformatics.psb.ugent.be/plaza/) and UniProt (http://www.uniprot.org).

### Predictor of intrinsic order and disorder

DISOPRED2 v2.42 [21] disorder predictions were performed for all protein sequences annotated in 12 plants, including proteins encoded in organelle genomes when available, and 22 bacteria. All input sequences, plus the reference database *uniref90*, were low-complexity filtered with PFILT and scanned with 3 iterations of *blastpgp* with an E-value cutoff of 0.001.

### A limited benchmark of disorder predictions in plant proteins

A computational experiment was carried out to estimate the quality of DISOPRED2 disorder predictions with plant protein sequences. The proteome of *A. thaliana* was compared to the contents of the Protein Data Bank as of February 7, 2012, looking for related structures. A total number of 70 crystallographic structures with ≥70% of sequence identity and resolution ≤2 Å were retrieved and used as a gold standard. Putative disordered segments of at least 30 residues were validated if aligned to residues reported in SEQRES records but absent in ATOM records, following the approach of the DISOPRED developers [20].

### Gene ontology (GO) analysis

Perl module GO::TermFinder v0.86, obtained from CPAN (http://search.cpan.org/dist/GO-TermFinder/), was used to estimate the enrichment in GO terms associated to sets of disordered proteins. GO mappings for all 12 proteomes were obtained from PLAZA and enrichments calculated with default parameters, with a false discovery rate of 1%. It must be noted that GO annotations retrieved from PLAZA for most genomes contained obsolete GO terms. The exact numbers found with respect to the official gene_ontology.1_2.obo release were: *A. thaliana* (350), *C. papaya* (0), *C. reindhartii* (1405), *O. sativa* (2824), *P. trichocarpa* (5200), *P. patens* (3055), *S. bicolor* (1814), *V. vinifera* (1491), *G. max* (539), *Micromonas sp.* RCC299 (49), *O. tauri* (35) and *Z. mays* (344).

## Abbreviations

acc, Acetyl CoA carboxylase; atp, ATP synthase; chl, Protochlorophyllide; fts, Penicillin binding protein, putative cell/organelle division protein; inf, Translational initiation factor; men, Succinyl-benzoate, succinyl-carboxilate and naphtoate synthase enzymes; ndh, NADH-plastoquinone oxidoreductase; pet, Cytochrome *b*_*6*_*/f* complex; psa, Photosystem I subunits; psb, Photosystem II subunits; rpl, Ribosomal L-proteins; rps, Ribosomal S-proteins; ribo, Ribosomal L-proteins plus ribosomal S-proteins.

## Competing interests

The authors declare that they have no competing interests.

## Authors’ contributions

IY carried out the sequence analysis, participated in the design and coordination of the study, and wrote the manuscript. BC-M participated in the design of the study and the data analysis, and helped write the manuscript. Both authors have read and approved the final manuscript.

## Supplementary Material

Additional file 1 **Table S1.**Distribution of predicted to-be-disordered segments with L ≥ 30, L ≥ 40 and L ≥ 50 in chloroplast, mitochondrial and nuclear plant proteomes. Click here for file

Additional file 2 **Table S2.**Statistical comparison of disorder content using Chi square tests (A) and Student's t tests (B). Click here for file

Additional file 3 **Figure S1.**Distribution of amino acid residues in disordered proteins in the plant proteomes. Nuclear (A), chloroplast (B), and mitochondrial (C) proteomes. Click here for file

Additional file 4 **Table S3.**Nucleus-encoded proteins with a putative orthologous copy in the chloroplast from *Arabidopsis thaliana*. ^1^ATC refers to proteins encoded by chloroplast genes. Click here for file

Additional file 5 **Table S4.**Selection results for Gene Ontology (GO) categories in intrinsically disordered proteins encoded by chloroplast genes and transferred to nuclear genome. A) biological process (P) GO categories; B) cellular components (C) GO categories; C) molecular function (F) GO categories. Click here for file

Additional file 6 **Table S5.**Selection results for gene ontology categories in non-disordered proteins encoded by chloroplast genes and transferred to nuclear genome. A) biological process (P) GO categories; B) cellular components (C) GO categories; C) molecular function (F) GO categories. Click here for file

Additional file 7 **Table S6.**Distribution of intrinsically disordered proteins in small (A) and large (B) ribosomal subunits from bacteria and plant chloroplast. Click here for file

Additional file 8 **Table S7.**Protein length of ribosomal proteins from bacteria and plant chloroplasts. Click here for file

## References

[B1] Dunker AK Obradovic Z Romero P Garner EC Brown CJ Intrinsic protein disorder in complete genomes Genome Inform 2000 11 161 171 11700597

[B2] Schlessinger A Schaefer C Vicedo E Schmidberger M Punta M Rost B Protein disorder – a breakthrough invention of evolution? Curr Opin Struct Biol 2011 21 412 418 10.1016/j.sbi.2011.03.014 21514145

[B3] Dyson HJ Wright PE Intrinsically unstructured proteins and their functions Nat Rev Mol Cell Biol 2005 6 197 208 10.1038/nrm1589 15738986

[B4] Radivojac P Obradovic Z Smith DK Zhu G Vucetic S Brown CJ Lawson JD Dunker AK Protein flexibility and intrinsic disorder Protein Sci 2004 13 71 80 10.1110/ps.03128904 14691223 PMC2286519

[B5] Tompa P Intrinsically unstructured proteins. Trends Biochem Sci 2002 27 527 533 10.1016/s0968-0004(02)02169-2 12368089

[B6] Radivojak P Iakoucheva LM Oldfield CJ Obradovic Z Uversky VN Dunker AK Intrinsic disorder and functional proteomics Biophys J 2007 92 1439 1456 10.1529/biophysj.106.094045 17158572 PMC1796814

[B7] Iakoucheva LM Brown CJ Lawson JD Obradovic Z Dunker AK Intrinsic disorder in cell-signaling and cancer-associated proteins J Mol Biol 2002 323 573 584 10.1016/S0022-2836(02)00969-5 12381310

[B8] Dedmon MM Patel CN Young GB Pielak GJ FlgM gains structure in living cells Proc Natl Acad Sci, USA 2002 99 12681 12684 10.1073/pnas.202331299 12271132 PMC130520

[B9] Dunker AK Brown CJ Lawson JD Iakoucheva LM Obradovic Z Intrinsic disorder and protein function Biochemistry 2002 41 6573 6582 10.1021/bi012159+ 12022860

[B10] Tompa P The interplay between structure and function in intrinsically unstructured proteins FEBS Lett 2005 579 3346 3354 10.1016/j.febslet.2005.03.072 15943980

[B11] Dunker AK Silman I Uversky VN Sussman JL Function and structure of inherently disordered proteins Curr Opin Struct Biol 2008 18 756 764 10.1016/j.sbi.2008.10.002 18952168

[B12] Van Roey K Gibson TJ Davey NE Motif switches: decision-making in cell regulation Curr Opin Struct Biol 2012 22 1 8 10.1016/j.sbi.2012.01.001 22480932

[B13] Kovacs D Kalmar E Torok Z Tompa P Chaperone activity of ERD10 and ERD14, two disordered stress-related plant proteins Plant Physiol 2008 147 381 390 10.1104/pp.108.118208 18359842 PMC2330285

[B14] Kovacs D Agoston B Tompa P Disordered plant LEA proteins as molecular chaperones Plant Signaling and Behaviour 2008 3 710 713 10.4161/psb.3.9.6434 PMC2634567 19704836

[B15] Mouillon J-M Eriksson SK Harryson P Mimicking the plant cell interior under water stress by macromolecular crowding: disordered dehydrin proteins Are highly resistant to structural collapse Plant Physiol 2008 148 1925 1937 10.1104/pp.108.124099 18849483 PMC2593683

[B16] Martin W Rujan T Richly E Hansen A Cornelsen S Lins T Leister D Stoebe B Hasegawa M Penny D Evolutionary analysis of Arabidopsis, cyanobacterial and chloroplast genomes reveals plastid phylogeny and thousands of cyanobacterial genes in the nucleus Procc Natl Acad Sci, USA 2002 99 12246 12251 10.1073/pnas.182432999 PMC129430 12218172

[B17] Dostányi Z Meszárós B Simon I Bioinformatical approaches to characterize intrinsically disordered/unstructured proteins Brief Bioinform 2010 11 225 243 10.1093/bib/bbp061 20007729

[B18] Linding R Jensen LJ Diella F Bork P Gibson TJ Russell RB Protein disorder prediction: implications for structural proteomics Structure 2003 11 1453 1459 10.1016/j.str.2003.10.002 14604535

[B19] Linding R Russell RB Neduva V Gibson TJ GlobPlot: exploring protein sequences for globularity and disorder Nucl. Acids Res 2003 31 3701 3708 10.1093/nar/gkg519 12824398 PMC169197

[B20] Ward JJ Sodhi JS McGuffin LJ Buxton BF Jones DT Prediction and functional analysis of native disorder in proteins from the three kingdoms of life J Mol Biol 2004 337 635 645 10.1016/j.jmb.2004.02.002 15019783

[B21] Ward JJ McGuffin LJ Bryson K Buxton BF Jones DT The DISOPRED server for the prediction of protein disorder Bioinformatics 2004 20 2138 2139 10.1093/bioinformatics/bth195 15044227

[B22] Dostányi Z Csizmok V Tompa P Simon I IUPred: web server for the prediction of intrinsically unstructured regions of proteins based on estimated energy content Structural Bioinformatics 2005 21 3433 3434 10.1093/bioinformatics/bti541 15955779

[B23] Romero P Obradovic Z Dunker AK Sequence data analysis for long disordered regions prediction in the calcineurin family Genome Inform 1997 8 110 124 11072311

[B24] Romero P Obradovic Z Li X Garner EC Brown CJ Dunker AK Sequence complexity of disordered protein Proteins 2001 42 38 48 10.1002/1097-0134(20010101)42:1<38::AID-PROT50>3.0.CO;2-3 11093259

[B25] Obradovic Z Peng K Vucetic S Radivojac P Brown CJ Dunker AK Predicting intrinsic disorder from amino acid sequence Proteins: Structure Function and Genetics 2003 53 566 572 10.1002/prot.10532 14579347

[B26] Yumi J Roland L Dunbrack RL Jr Assessment of disorder predictions in CASP6 Proteins: Structure, Function, and Bioinformatics 2005 61 167 175 10.1002/prot.20734 16187359

[B27] Monastyrskyy B Fidelis K Moult J Tramontano A Kryshtafovych A Evaluation of disorder predictions in CASP9 Proteins 2011 10 107 118 21928402 10.1002/prot.23161 PMC3212657

[B28] Mueller F Sommer I Baranov P Matadeen R Stoldt M Woehnert J Goerlach M van Heel M Brimacombe R The 3D arrangement of the 23S and 5S rRNA in the Escherichia coli 50S ribosomal subunit based on a cryo-electron microscopic reconstruction at 7.5 Å resolution J Mol Biol 2000 298 35 59 10.1006/jmbi.2000.3635 10756104

[B29] Gao H Sengupta J Valle M Korostelev A Eswar N Stagg SM Van Roey P Agrawal RK Harvey SC Sali A Chapman MS Frank J Study of the structural dynamics of the E coli 70S ribosome using real-space refinement Cell 2003 113 789 801 10.1016/S0092-8674(03)00427-6 12809609

[B30] Merianos HJ Wang J Moore PB The structure of a ribosomal protein S8/spc operon mRNA complex RNA 2004 10 954 964 10.1261/rna.7030704 15146079 PMC1370587

[B31] Lobanov MY Furletova EI Bogatyreva NS Roytberg MA Galzitskaya OV Library of disordered patterns in 3D protein strutures PLoS Comput Biol 2010 6 10 e1000958 10.1371/journal.pcbi.1000958 20976197 PMC2954861

[B32] Abdallah F Salamini F Leister D A prediction of the size and evolutionary origin of the proteome of chloroplasts of Arabidopsis Trends Plant Sci 2000 5 141 142 10.1016/S1360-1385(00)01574-0 10928822

[B33] Cavalier-Smith T Membrane heredity and early chloroplast evolution Trends Plant Sci 2000 5 174 182 10.1016/S1360-1385(00)01598-3 10740299

[B34] Rujan T Martin W How many genes in Arabidopsis come from cyanobacteria? An estimate from 386 protein phylogenies Trends Genet 2001 17 113 120 10.1016/S0168-9525(00)02209-5 11226586

[B35] Stegemann S Hartmann S Ruf S Bock R High-frequency gene transfer from the chloroplast genome to the nucleus Proc Natl Acad Sci, USA 2003 100 8828 8833 10.1073/pnas.1430924100 12817081 PMC166398

[B36] Rechsteiner M Rogers SW PEST sequences and regulation by proteolysis Trends Biochem Sci 1996 21 267 271 8755249

[B37] Sekhar KR Freeman ML PEST sequences in proteins involved in cyclic nucleotide signalling pathways Journal of Receptors and Signal Transduction Research 1998 18 113 132 10.3109/10799899809047740 9651881

[B38] Ban N Nissen P Hansen J Moore P Steitz TA The complete atomic structure of the large ribosomal subunit at 2.4 Å resolution Science 2000 289 905 920 10.1126/science.289.5481.905 10937989

[B39] DiNitto JP Huber PW Mutual induced fit binding of Xenopus ribosomal protein L5 to 5S-rRNA J Mol Biol 2003 330 979 992 10.1016/S0022-2836(03)00685-5 12860121

[B40] Worbs M Huber R Wahl MC Crystal structure of ribosomal protein L4 shows RNA-binding sites for ribosome incorporation and feedback control of the S10 operon EMBO J 2000 19 807 818 10.1093/emboj/19.5.807 10698923 PMC305621

[B41] Semrad K Green R Schroeder R RNA chaperone activity of large ribosomal subunit proteins from Escherichia coli RNA 2004 10 1855 1860 10.1261/rna.7121704 15525706 PMC1370674

[B42] Yamaguchi K Subramanian AR The plastid ribosomal proteins. Identification of all the proteins in the 50S subunit of an organelle ribosome (chloroplast) J Biol Chem 2000 275 28466 28482 10874046 10.1074/jbc.M005012200

[B43] Harris EH Boynton JE Gillham NW Chloroplast ribosomes and protein synthesis Microbiol Rev 1994 58 700 754 7854253 10.1128/mr.58.4.700-754.1994 PMC372988

[B44] Chi W He B Mao J Li Q Ma J Ji D Zou M Zhang L The Function of RH22, a DEAD RNA Helicase, in the Biogenesis of the 50S Ribosomal Subunits of Arabidopsis Chloroplasts Plant Physiol 2012 158 693 707 10.1104/pp.111.186775 22170977 PMC3271760

[B45] Martin W Herrmann RG Gene transfer from organelles to the nucleus: how much, what happens, and why? Plant Physiol 1998 118 9 17 10.1104/pp.118.1.9 9733521 PMC1539188

[B46] Sharma MR Wilson DN Datta PP Barat C Schluenzen F Fucini P Agrawal RK Cryo-EM study of the spinach chloroplast ribosome reveals the structural and functional roles of plastid-specific ribosomal proteins Proc Natl Acad Sci, USA 2007 104 19315 19320 10.1073/pnas.0709856104 18042701 PMC2148287

