# Supplementary material for: Protein disorder in plants: a view from the chloroplast
Source: BMC Plant Biol. 2012 Sep 13;12:165. doi: 10.1186/1471-2229-12-165 (PMC3460767; doi:10.1186/1471-2229-12-165)
Supplement: Additional file 1 — Table S1.Distribution of predicted to-be-disordered segments with L ≥ 30, L ≥ 40 and L ≥ 50 in chloroplast, mitochondrial and nuclear plant proteomes. [file 1471-2229-12-165-S1.pdf]

Table S1.- Disorder in plant proteomes

Proteomes	Chloroplasts				Mitochondria				Nuclear			
	N proteins	L >= 30	L >= 40	L >= 50	N proteins	L >= 30	L >= 40	L >= 50	N proteins	L >= 30	L >= 40	L >= 50
**Vascular plants**												
**Eudicots/Rosids/Brassicales**												
Arabidopsis thaliana	87	9.19	3.45	0.00	117	19.49	13.56	6.70	22,175	52.65	40.91	32.79
Carica papaya	84	7.06	2.35	1.18	-	nd	nd	nd	22,067	43.73	33.03	26.06
**Eudicots/Rosids/Fabales**												
Glycine max	82	6.02	2.41	1.20	-	nd	nd	nd	28,530	53.28	42.17	30.64
**Eudicots/Rosids/Malpighiales**												
Populus trichocarpa	100	10.89	3.96	1.98	-	nd	nd	nd	38,835	40.21	30.18	23.57
Eudicots/Rosids/Vitales												
Vitis vinifera	86	4.60	2.30	0.00	74	14.67	2.67	2.67	30,534	40.11	29.85	23.12
**Monocots**												
Oryza sativa	108	7.41	1.85	0.93	53	18.52	7.41	7.41	38,227	49.35	36.83	27.96
Sorghum bicolor	84	7.06	3.53	1.17	32	12.12	6.06	6.06	32,219	50.79	40.06	32.10
Zea mays	99	6.31	2.70	1.80	164	17.57	9.70	7.27	22,271	56.17	44.39	31.00
**Bryophyta**												
Physcomitrella_patens	85	5.81	3.49	1.16	42	6.98	4.65	0.00	34,271	38.23	26.72	19.48
**Chlorophyta**												
Chlamydomonas reindhardtii	69	12.86	10.00	4.29	14	6.67	6.67	6.67	13,201	45.42	33.79	25.45
Micromonas sp RCC299	56	1.75	0.00	0.00	38	10.25	0.00	0.00	8,989	52.91	39.61	29.92
Ostreococcus tauri	60	4.92	1.64	1.64	42	2.32	0.00	0.00	7,396	52.52	38.08	27.43
