# Supplementary material for: Protein disorder in plants: a view from the chloroplast
Source: BMC Plant Biol. 2012 Sep 13;12:165. doi: 10.1186/1471-2229-12-165 (PMC3460767; doi:10.1186/1471-2229-12-165)
Supplement: Additional file 3 — Figure S1.Distribution of amino acid residues in disordered proteins in the plant proteomes. Nuclear (A), chloroplast (B), and mitochondrial (C) proteomes. [file 1471-2229-12-165-S3.pdf]

Figure S1. Distribution of amino acid residues in disordered proteins in the plant proteomes. Nuclear (A), chloroplast (B), and mitochondrial (C) proteomes.

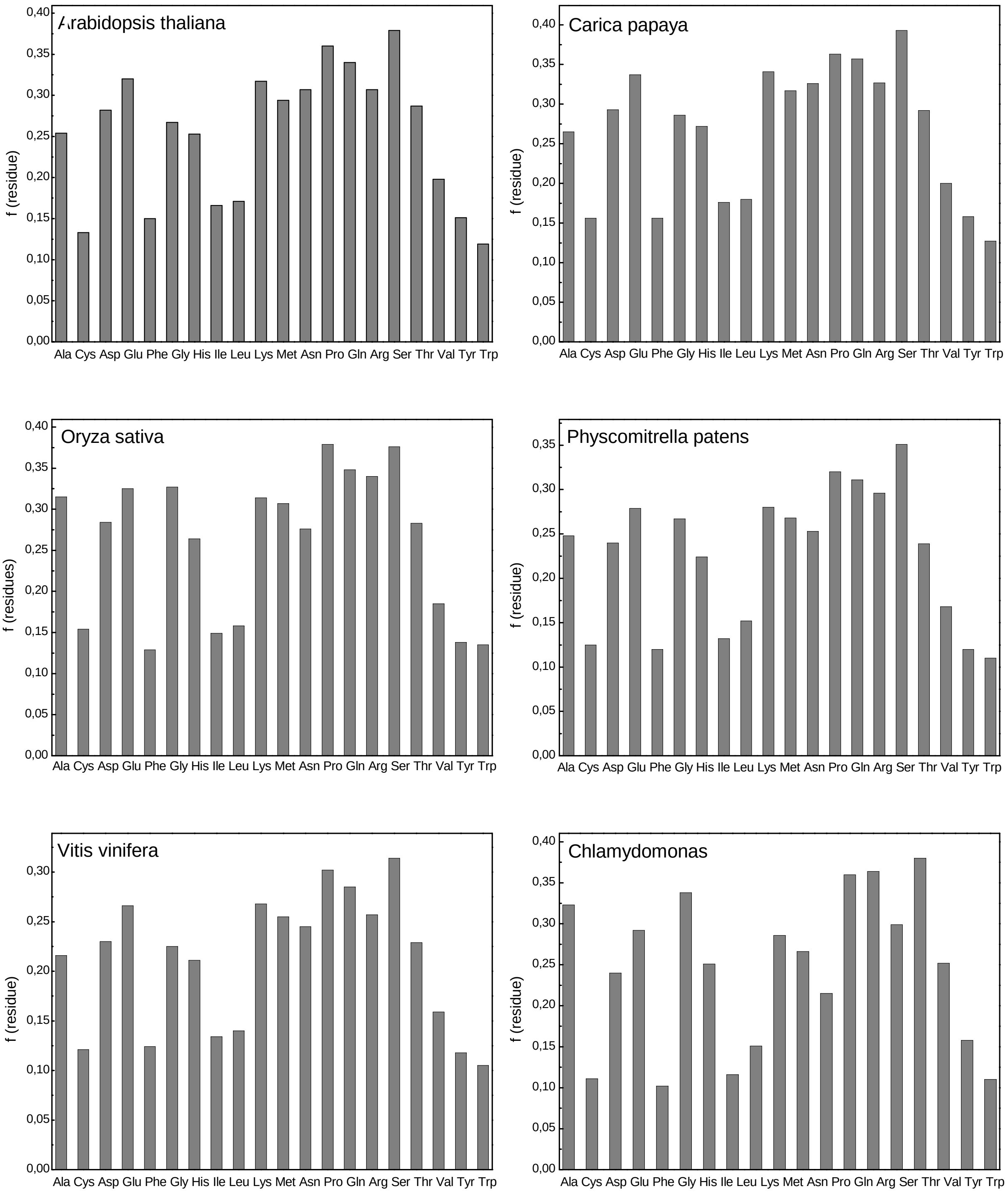

NUCLEAR PROTEOME

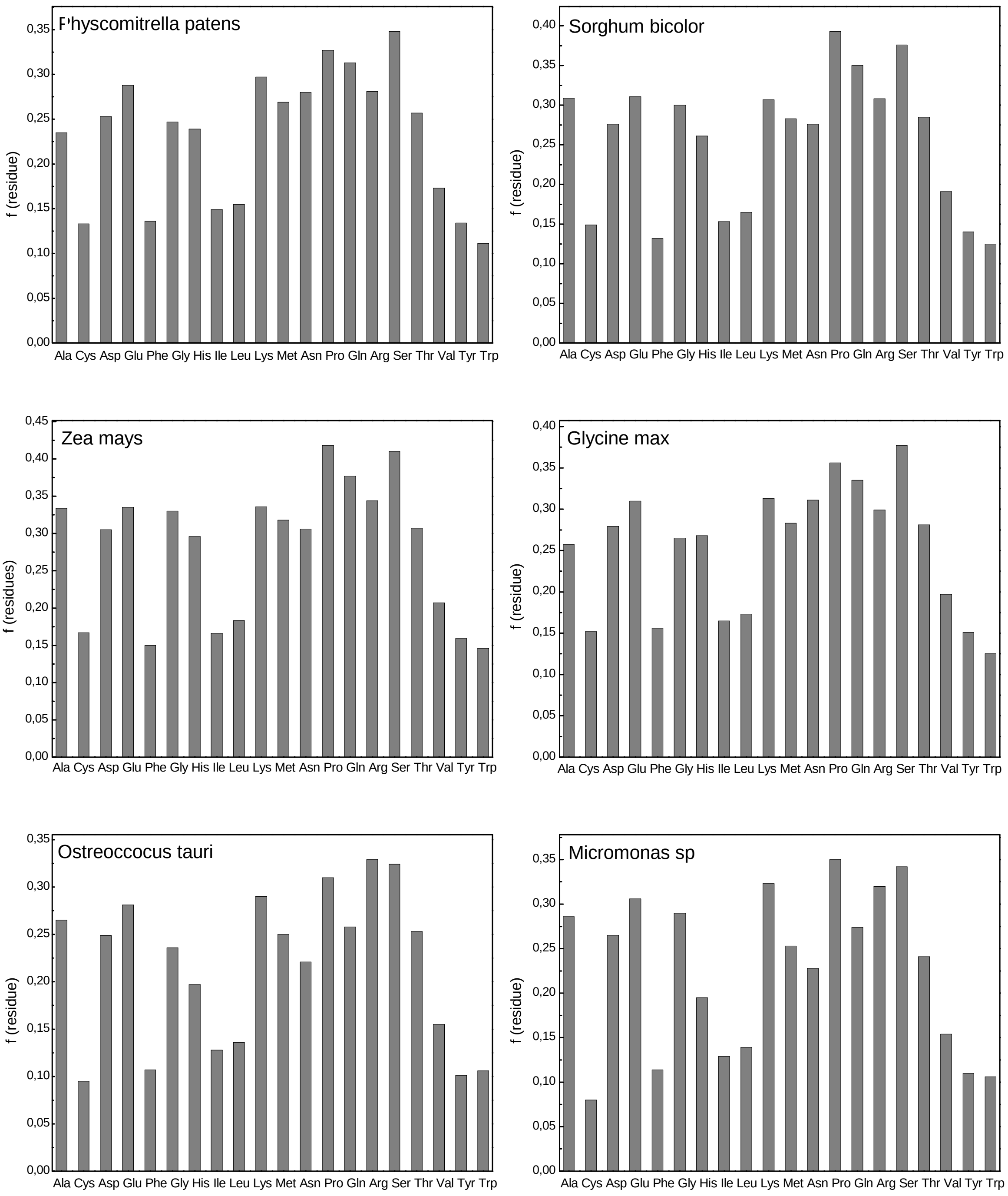

NUCLEAR PROTEOME

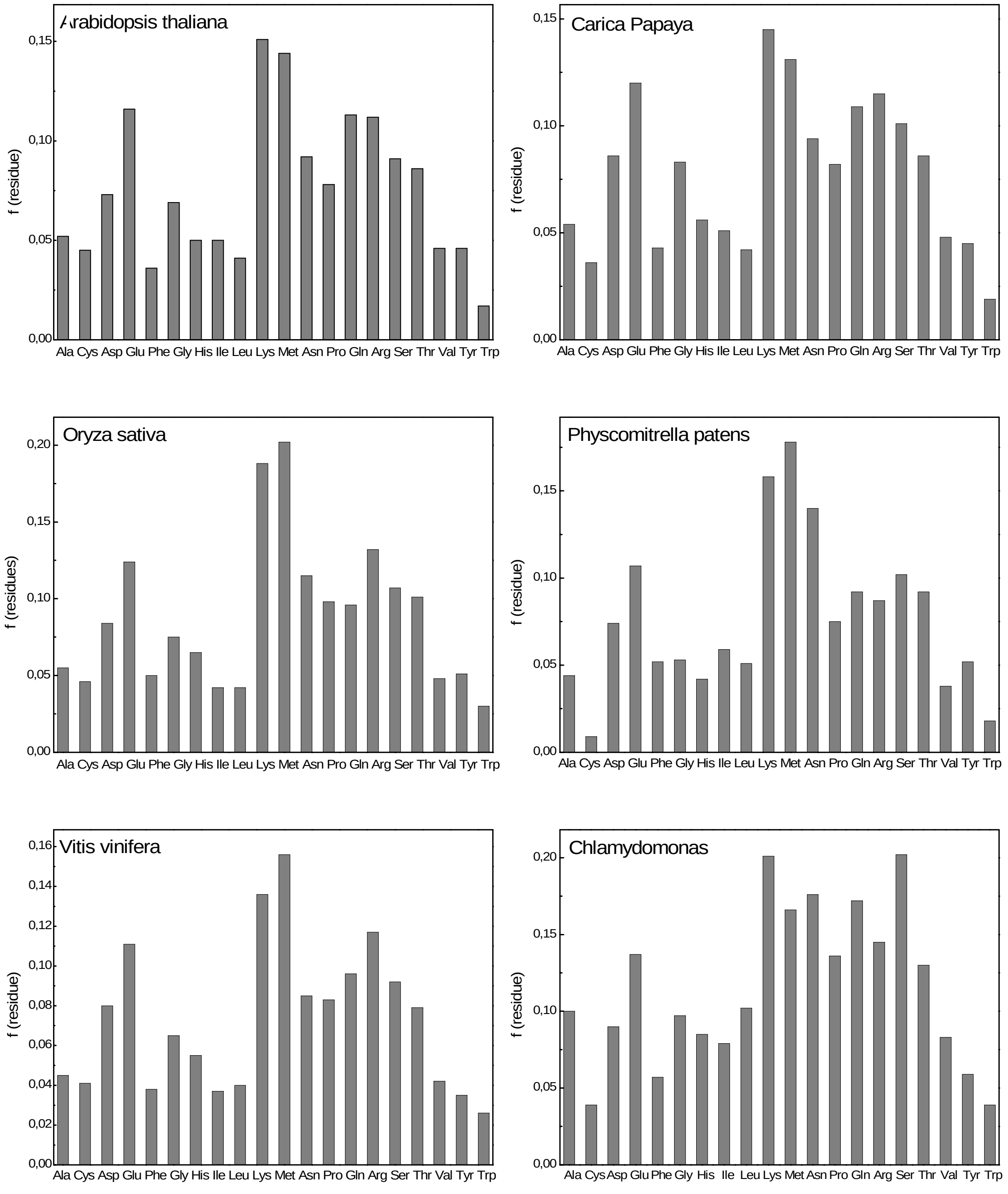

CHLOROPLAST PROTEOME

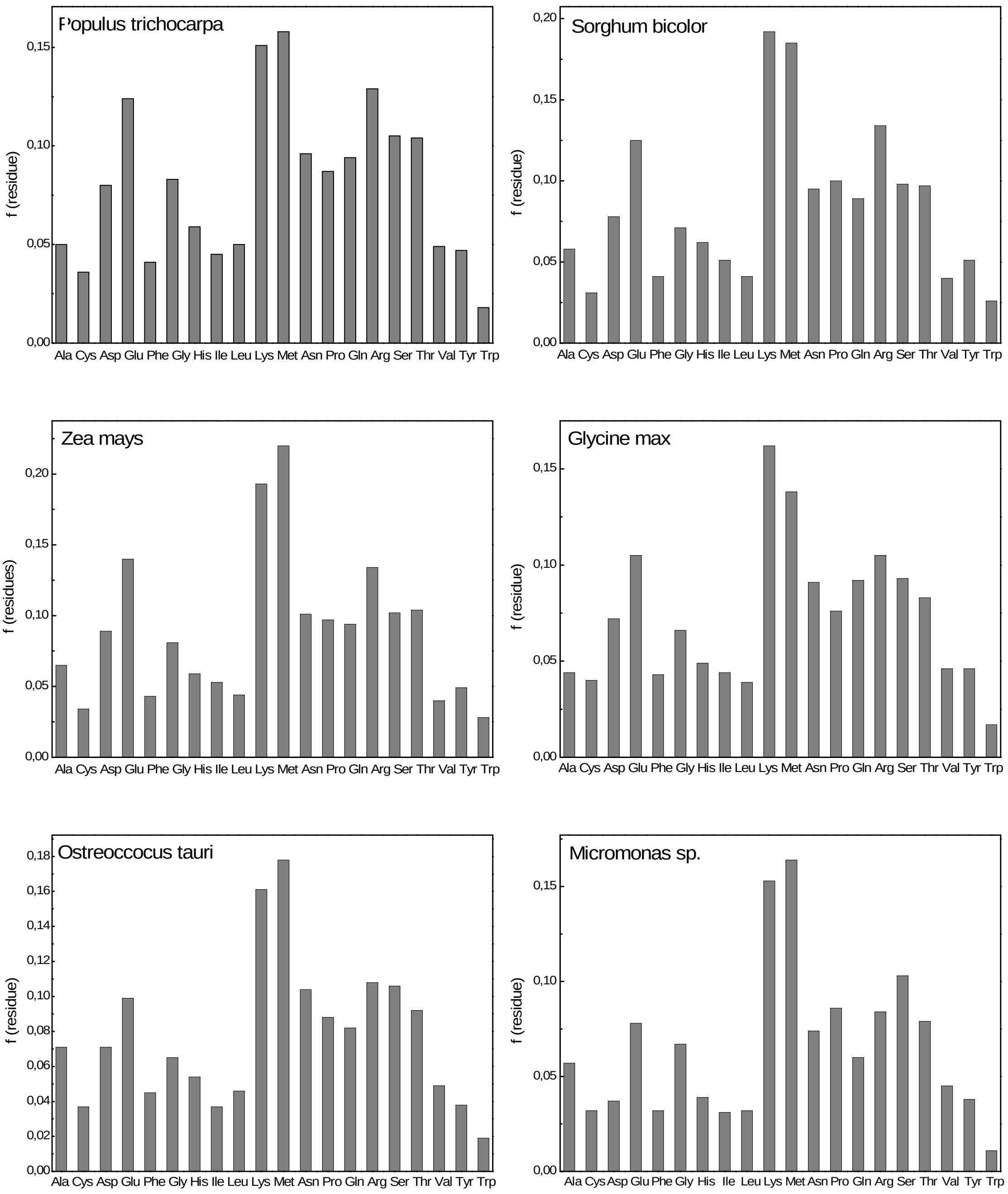

CHLOROPLAST PROTEOME

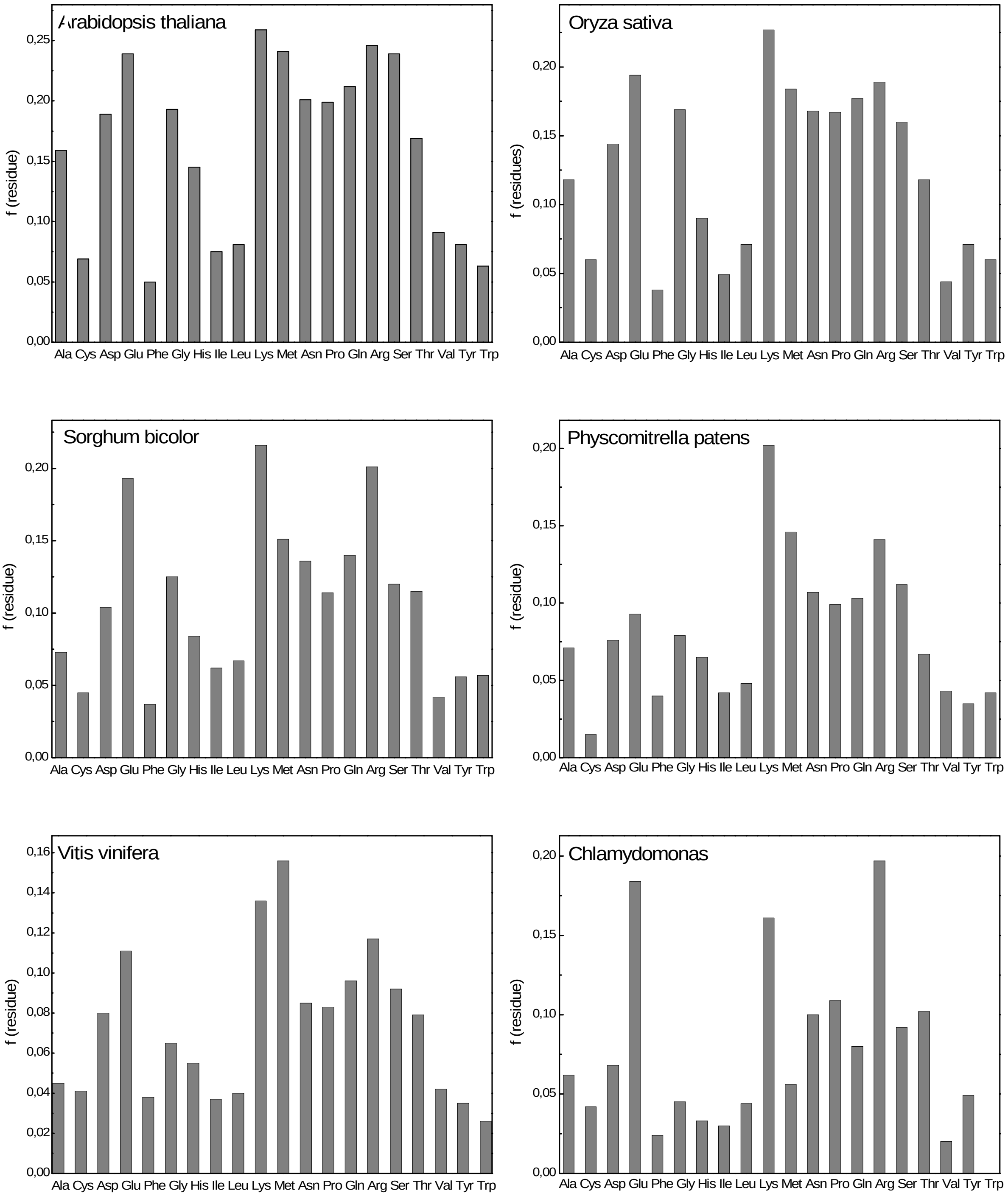

MITOCHONDRIAL PROTEOME

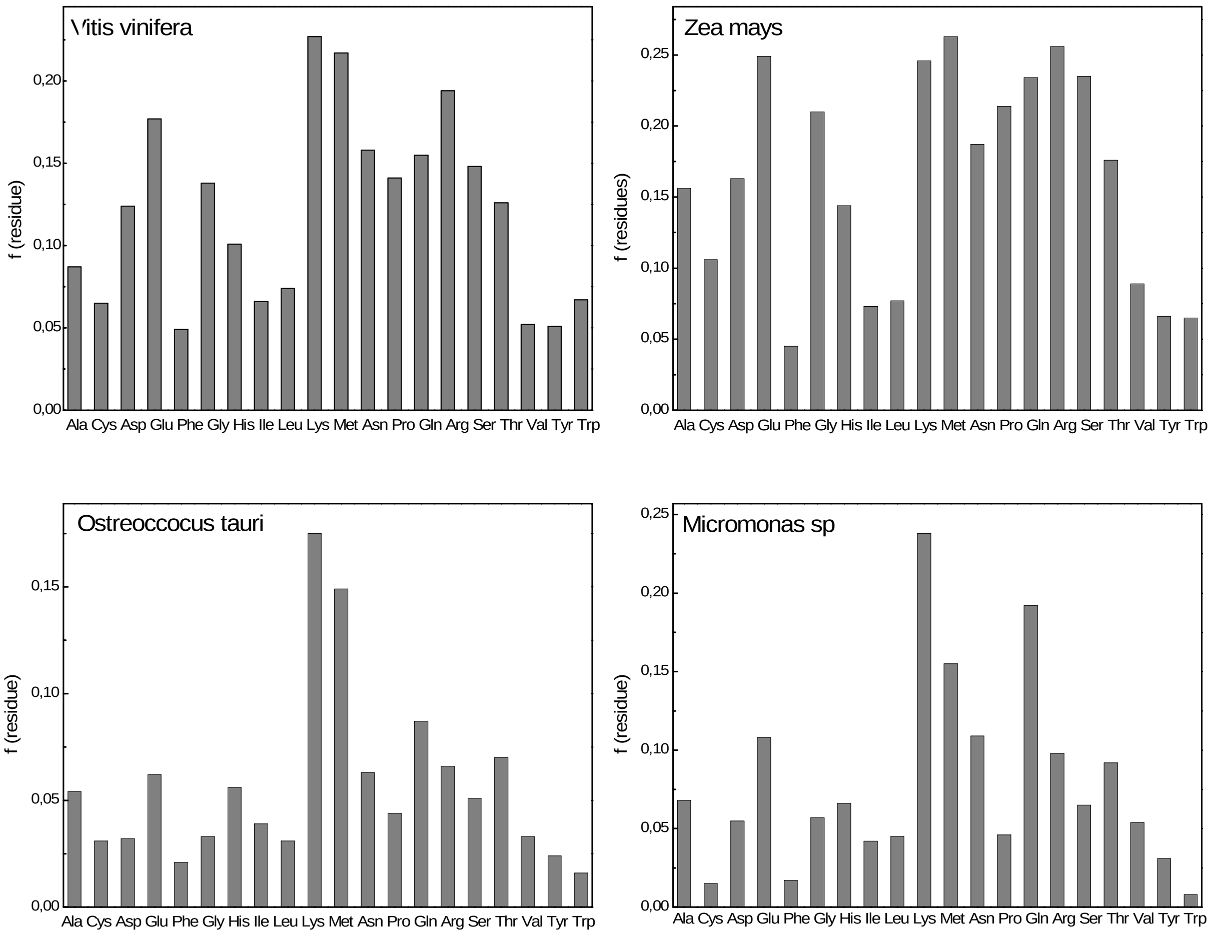

MITOCHONDRIAL PROTEOME
