# Supplementary material for: Protein disorder in plants: a view from the chloroplast
Source: BMC Plant Biol. 2012 Sep 13;12:165. doi: 10.1186/1471-2229-12-165 (PMC3460767; doi:10.1186/1471-2229-12-165)
Supplement: Additional file 4 — Table S3.Nucleus-encoded proteins with a putative orthologous copy in the chloroplast from Arabidopsis thaliana. 1ATC refers to proteins encoded by chloroplast genes. [file 1471-2229-12-165-S4.pdf]

Table S7.- Nucleus-encoded proteins with a putative orthologous copy in the chloroplast from *Arabidopsis thaliana.* [1]ATC refers to proteins encoded by chloroplast genes.

Name	ID	Length	IDP (L ≥ 30)	Disordered segments (L ≥ 30)
ATP synthase alpha subunit, atpA	AT2G07698<br>[1]ATCG00120	777<br>507	-<br>-	
ATP synthase subunit B1, atpB	AT1G20260<br>ATCG00480	487<br>498	-<br>-	
ATP synthase subunit A1, atpI	AT2G07741<br>ATCG00150	385<br>249	-<br>-	
CemA	AT4G31040<br>ATCG00530	438<br>438	+<br>-	16-48; 151-181
NADH Ubiquinone/plastoquinone (complex I), ndhB	AT2G07689<br>ATCG00890<br>ATCG01250	214<br>389<br>389	-<br>-<br>-	
NADH dehydrogenase subunit 3, ndhC	AT2G07751<br>ATCG00440	118<br>120	-<br>-	
NADH dehydrogenase [ubiquinone] iron-sulfur protein 8, ndhG	AT1G79010<br>AT1G16700<br>ATCG01100	222<br>222<br>360	-<br>-<br>-	
NADH dehydrogenase (ubiquinone), ndhI	AT2G07785<br>ATCG01100	99<br>360	-<br>-	
Citochrome b, petB	AT2G07727<br>ATCG00720	393<br>215	-<br>-	
Ribulose-bisphosphate carboxylase, rbcLg	AT2G07732<br>ATCG00490	116<br>479	-<br>-	
Ribosomal protein L2	AT2G44065<br>ATCG00830<br>ATCG01310	214<br>274<br>274	-<br>+<br>+	 230-265<br>230-265
Ribosomal protein L14p/L23e	AT5G46160<br>AT1G17560<br>AT2G33370<br>AT1G04480<br>AT3G04400<br>ATCG00780	173<br>196<br>140<br>140<br>140<br>122	-<br>-<br>-<br>-<br>-<br>-	
Ribosomal protein L20	AT1G16740<br>AT4G09468<br>ATCG00660	126<br><br>117	-<br>-<br>-	
Ribosomal protein L22p/L17e	AT1G52370<br>AT4G28360<br>AT1G27400<br>AT1G67430<br>ATCG00810	269<br>271<br>176<br>175<br>160	-<br>-<br>-<br>-<br>-	
Ribosomal protein L36	AT5G20180<br>ATCG00760	103<br>37	-<br>-	
Subunit of RNA polymerase complex, rpoB	AT4G21710<br>AT1G29940<br>AT3G18090<br>AT3G23780<br>AT5G45140<br>ATCG00190	1188<br>1178<br>1055<br>1172<br>1161<br>1072	-<br>-<br>-<br>-<br>-<br>-	
Subunit of RNA polymerase complex, rpoC	AT5G60040<br>ATCG00180<br>ATCG00170	1391<br>680<br>1376	-<br>-<br>-	

Ribosomal protein S15ae / S8	AT4G29430	129	-	
	AT3G46040	130	-	
	AT1G07770	130	-	
	AT2G39590	136	-	
	AT5G59850	130	-	
	AT2G19720	129	-	
	ATCG00770	134	-	
Ribosomal protein S14	AT2G34520	164	-	
	ATCG00330	100	-	
Ribosomal protein S16	AT4G34620	113	+	85-113
	AT5G56940	135	+	86-135
	ATCG00050	79	-	
Ribosomal protein S19 /S15	AT5G63070	160	-	
	AT5G09490	152	-	
	AT5G43640	149	-	
	AT5G09500	150	-	
	AT5G09510	152	-	
	AT1G04270	152	-	
	ATCG00820	92	-	
Ribosomal protein L23	AT4G39880	178	-	
	ATCG00840	93	-	
	ATCG01300	93		
ycf1 protein	AT2G07739	199	-	
	ATCG01000	343	-	
