# Supplementary material for: Protein disorder in plants: a view from the chloroplast
Source: BMC Plant Biol. 2012 Sep 13;12:165. doi: 10.1186/1471-2229-12-165 (PMC3460767; doi:10.1186/1471-2229-12-165)
Supplement: Additional file 5 — Table S4.Selection results for Gene Ontology (GO) categories in intrinsically disordered proteins encoded by chloroplast genes and transferred to nuclear genome. A) biological process (P) GO categories; B) cellular components (C) GO categories; C) molecular function (F) GO categories. [file 1471-2229-12-165-S5.pdf]

Table S2_A.- Selection results for P-branch gene ontology (GO) categories in intrinsically disordered proteins encoded by chloroplast genes and transferred to nuclear genome.

GO annotation	AT	CP	PT	VV	OS	SB	ZM	GM	PP
cellular biosynthetic process	53 / 98<br>1.07 E-23	18 / 30<br>4.52 E-10	37 / 63<br>6.31 E-18	34 / 67<br>1.45 E-14	27 / 54<br>3.03 E-08	34 / 68<br>3.22 E-16	-	17 / 30<br>8.17 E-09	28 / 55<br>8.59 E-10
biosynthetic process	53 / 98<br>3.97 E-22	18 / 30<br>2.54 E-09	40 / 63<br>1.36 E-19	35 / 67<br>7.27 E-14	33 / 54<br>4.27 E-09	37 / 68<br>3.43 E-17	-	18 / 30<br>3.40 E-09	30 / 55<br>4.04 E-10
small molecule biosynthetic process	-	11 / 30<br>9.73 E-09	20 / 63<br>2.25 E-13	16 / 67<br>3.09 E-09	9 / 54<br>7.07 E-06	13 / 68<br>7.56 E-07	-	-	-
cellular metabolic process	66 / 98<br>2.45 E-16	20 / 30<br>5.12 E-06	-	-	41 / 54<br>9.40 E-08	-	-	22 / 30<br>8.44 E-06	-
metabolic process	72 / 98<br>2.53 E-14	-	50 / 63<br>2.89 E-07	-	45 / 54<br>2.23 E-06	52 / 68<br>1.65 E-06	34 / 44<br>3.54 E-07	-	-
cellular nitrogen compound biosynthetic process	20 / 98<br>1.10 E-13	-	14 / 63<br>1.07 E-07	12 / 67<br>4.80 E-06	-	12 / 68<br>4.77 E-06	-	-	-

cellular process	74 / 98 1.18 E-13	-	-	-	47 / 54 3.20 E-07	-	-	-	-
cofactor biosynthetic process	14 / 98 4.20 E-13	-	8 / 63 1.37 E-06	-	-	7 / 68 2.48 E-06	-	-	-
translation	22 / 98 6.91 E-13	-	-	-	15 / 54 9.75 E-09	15 / 68 6.36 E-07	-	11 / 30 1.83 E-08	17 / 55 4.70 E-07
heterocycle biosynthetic process	14 / 98 4.68 E-10	-	-	-	-	10 / 68 6.84 E-06	-	-	-
cofactor metabolic process	-	-	-	-	-	9 / 68 1.45 E-07	-	-	-
tetrapyrrole biosynthetic process	8 / 98 9.77 E-09	-	-	-	-	-	-	-	-
gene expresión	23 / 98 1.64 E-08	-	-	-	-	-	-	11 / 30 2.81 E-06	-
cellular amino acid biosynthetic process	11 / 98 2.11 E-10	-	-	-	-	-	-	-	-

**porphyrin metabolic process**	**8 / 98<br>5.51 E-08**	**-**	**-**	**-**	**-**	**6 / 68<br>9.57 E-07**	**-**	**-**	**-**
**tetrapyrrole metabolic process**	**8 / 98<br>6.33 E-08**	**-**	**-**	**-**	**-**	**6 / 68<br>1.13 E-06**	**-**	**-**	**-**
**organic acid biosynthetic process**	**14 / 98<br>1.18 E-07**	**9 / 30<br>1.99 E-07**	**14 / 63<br>8.58 E-10**	**12 / 67<br>1.51 E-07**	**-**	**-**	**-**	**-**	**-**
**carboxylic acid biosynthetic process**	**14 / 98<br>5.67 E-10**	**9 / 30<br>1.99 E-07**	**14 / 63<br>8.58 E-10**	**12 / 67<br>1.51 E-07**	**-**	**-**	**-**	**-**	**-**
**amine biosynthetic process**	**11 / 98<br>1.27 E-07**	**-**	**-**	**-**	**-**	**-**	**-**	**-**	**-**
**porphyrin biosynthetic process**	**7 / 98<br>2.79 E-07**	**-**	**-**	**-**	**-**	**-**	**-**	**-**	**-**
**primary metabolic process**	**54 / 98<br>4.04 E-07**	**-**	**-**	**-**	**-**	**-**	**-**	**-**	**-**
**heterocycle metabolic process**	**14 / 98<br>5.05 E-07**	**-**	**-**	**-**	**13 / 68<br>6.31 E-08**	**-**	**-**	**-**	**-**

**cellular ketone metabolic process**	**17 / 98**<br>**1.48 E-06**	**10 / 30**<br>**1.72 E-06**	**16 / 63**<br>**4.58 E-08**	**-**	**-**	**-**	**-**	**-**	**-**
**pigment metabolic process**	**8 / 98**<br>**2.04 E-06**	**-**	**-**	**-**	**-**	**-**	**-**	**-**	**-**
**small molecule metabolic process**	**-**	**-**	**20 / 63**<br>**1.45 E-07**	**-**	**-**	**-**	**-**	**-**	**-**
**carboxylic acid metabolic process**	**-**	**-**	**14 / 63**<br>**3.21 E-06**	**-**	**-**	**-**	**-**	**-**	**-**
**oxoacid metabolic process**	**-**	**-**	**14 / 63**<br>**3.22 E-06**	**-**	**-**	**-**	**-**	**-**	**-**
**organic acid metabolic process**	**-**	**-**	**14 / 63**<br>**3.32 E-06**	**-**	**-**	**-**	**-**	**-**	**-**
**branched chain family amino acid biosynthetic process**	**-**	**-**	**4 / 63**<br>**6.40 E-06**	**-**	**4 / 54**<br>**1.20 E-06**	**-**	**-**	**-**	**-**

Table S2_B.- Selection results for C-branch gene ontology (GO) categories in intrinsically disordered proteins encoded by chloroplast genes and transferred to nuclear genome.

GO annotations	AT	CP	PT	VV	OS	SB	ZM	GM	PP
plastid	75 / 98<br>4.60 E-43	26 / 30<br>5.64 E-17	34 / 63<br>5.06 E-23	34 / 67<br>8.05 E-23	42 / 54<br>4.68 E-12	-	27 / 44<br>1.62 E-15	-	-
chloroplast	74 / 98<br>1.83 E-42	26 / 30<br>2.26 E-17	34 / 63<br>1.30 E-23	33 / 67<br>2.42E-22	-	-	-	-	-
cytoplasmic part	86 / 98<br>1.09 E-37	29 / 30<br>4.42 E-14	43 / 63<br>6.05 E-19	40 / 67<br>1.38 E-16	-	-	35 / 44<br>5.91 E-11	-	-
cytoplasm	87 / 98<br>1.30 E-36	29 / 30<br>3.90 E-13	43 / 63<br>2.92 E-17	41 / 67<br>8.66 E-16	-	-	35 / 44<br>2.75 E-10	19 / 30<br>3.02 E-06	-
organelle	-	-	-	-	-	-	35 / 44<br>6.90 E-08	-	-
plastid part	45 / 98<br>2.35 E-33	12 / 30<br>9.50 E-06	18 / 63<br>1.39 E-11	16 / 67<br>6.13 E-10	-	-	-	-	-

**chloroplast part**	**44 / 98**<br>**1.50 E-32**	**12 / 30**<br>**6.46 E-06**	**18 / 63**<br>**6.49 E-12**	**16 / 67**<br>**2.56 E-10**	**-**	**-**	**-**	**-**	**-**
**intracelular**	**89 / 98**<br>**3.54 E-25**	**29 / 30**<br>**1.40 E-09**	**43 / 63**<br>**1.52 E-06**	**41 / 67**<br>**9.49 E-07**	**-**	**-**	**35 / 44**<br>**2.64 E-06**	**-**	**-**
**plastid envelope**	**30 / 98**<br>**4.26 E-25**	**-**	**12 / 63**<br>**1.00 E-08**	**12 / 67**<br>**1.27 E-09**	**-**	**-**	**-**	**-**	**-**
**intracellular part**	**87 / 98**<br>**1.87 E-24**	**29 / 30**<br>**9.28 E-10**	**43 / 63**<br>**4.43 E-07**	**41 / 67**<br>**9.23 E-08**	**-**	**-**	**35 / 44**<br>**4.09 E-07**	**-**	**-**
**chloroplast envelope**	**29 / 98**<br>**2.88 E-24**	**-**	**12 / 63**<br>**6.85 E-09**	**12 / 67**<br>**8.25 E-10**	**-**	**-**	**-**	**-**	**-**
**intracellular membrane-bounded organelle**	**82 / 98**<br>**5.29 E-24**	**29 / 30**<br>**3.38 E-11**	**35 / 63**<br>**1.20 E-06**	**35 / 67**<br>**5.28 E-07**	**-**	**-**	**-**	**-**	**-**
**intracellular organelle**	**-**	**-**	**-**	**-**	**-**	**-**	**35 / 44**<br>**6.90 E-08**	**-**	**-**

**membrane-bounded organelle**	**82 / 98**<br>**5.35 E-24**	**29 / 30**<br>**3.43 E-11**	**35 / 63**<br>**1.26 E-06**	**35 / 67**<br>**5.31 E-07**	**-**	**-**	**-**	**-**	**-**
**plastid stroma**	**29 / 98**<br>**1.20 E-23**	**-**	**13 / 63**<br>**3.49 E-10**	**11 / 67**<br>**6.66 E-08**	**-**	**-**	**-**	**-**	**-**
**intracellular organelle part**	**52 / 98**<br>**1.47 E-23**	**-**	**41 / 6**<br>**5.61 E-09**	**39 / 67**<br>**4.57 E-08**	**-**	**-**	**-**	**-**	**-**
**organelle part**	**52 / 98**<br>**1.50 E-23**	**-**	**-**		**-**	**-**	**-**	**-**	**-**
**chloroplast stroma**	**28 / 98**<br>**7.62 E-23**	**-**	**13 / 63**<br>**1.05 E-10**	**11 / 67**<br>**1.93 E-08**	**-**	**-**	**-**	**-**	**-**
**organelle envelope**	**30 / 98**<br>**3.78 E-20**	**-**	**12 / 63**<br>**2.21 E-06**	**12 / 67**<br>**2.12 E-07**	**-**	**-**	**-**	**.**	**-**
**envelope**	**30 / 98**<br>**3.78 E-20**	**-**	**12 / 63**<br>**8.05 E-06**	**12 / 67**<br>**2.12 E-07**	**-**	**-**	**-**	**.**	**-**
**thylakoid**	**18 / 98**<br>**2.78 E-11**	**-**	**-**	**-**	**-**	**-**	**-**	**.**	**-**

ribosome	16 / 98<br>7.94 E-09	-	-	-	-		-	11 / 30<br>7.14 E-10	13 / 55<br>8.93 E-06
organellar ribosome	5 / 98<br>1.80 E-07	-	-	-	-	-	-	-	-
chloroplast thylakoid membrane	12 / 98<br>5.71 E-07	-	-	-	-	-	-	-	-
plastid thylakoid membrane	12 / 98<br>5.71 E-07	-	-	-	-	-	-	-	-
ribonucleo protein complex	16 / 98<br>7.77 E-07	.	-	-	-	-	-	11 / 30<br>1.90 E-09	-
thylakoid membrane	12 / 98<br>9.04 E-07	.	-	-	-	-	-	-	-
photosynthe-tic membrane	12 / 98<br>1.01 E-06	.	-	-	-	-	-	-	-

**cell**	**90 / 98**<br>**2.14 E-06**	**.**	**-**	**51 / 67**<br>**5.09 E-07**	**-**	**-**	**-**	**-**	**-**
**cell part**	**90 / 98**<br>**2.14 E-06**	**.**	**-**	**51 / 67**<br>**5.01 E-07**	**-**	**-**	**-**	**-**	**-**
**large ribosomal subunit**	**9 / 98**<br>**2.21 E-06**	**.**	**-**	**-**<br>**-**	**-**	**-**	**-**	**-**	**-**
**plastid ribosome**	**4 / 98**<br>**2.26E-06**	**.**	**-**	**-**	**-**	**-**	**-**	**-**	**-**
**plastid thylakoid**	**12 / 98**<br>**2.53 E-06**	**.**	**-**	**-**	**-**	**-**	**-**	**-**	**-**
**organelle subcompart-ment**	**12 / 98**<br>**2.71 E-06**	**.**	**-**	**-**	**-**	**-**	**-**	**-**	**-**
**thylakoid part**	**12 / 98**<br>**3.76 E-06**	**.**	**-**	**-**	**-**	**-**	**-**	**-**	**-**

**macromole-cular complex**	**-**	**-**	**-**	**-**	**-**	**-**	**14 / 30**<br>**2.02 E-08**	**-**	**-**
**non-membrane-bounded organelle**	**-**	**-**	**-**	**-**	**-**	**-**	**11 / 30**<br>**6.63 E-07**	**-**	**-**
**intracellular non-membranE-bounded organelle**	**-**	**-**	**-**	**-**	**-**	**-**	**11 / 30**<br>**6.63 E-07**	**.**	**-**

Table S2_C.- Selection results for F-branch gene ontology (GO) categories in intrinsically disordered proteins encoded by chloroplast genes and transferred to nuclear genome.

GO annotations	AT	CP	PT	VV	OS	ZM	GM	PP
structural constituent of ribosome	15 / 98 8.01 E-09	-	-	-	-	-	-	-
transferase activity, transferring aldehyde or ketonic groups	-	-	6 / 63 6.87 E-10	-	4 / 54 2.35E-07	-	-	-
structural molecule activity	16 / 98 8.18 E-08	-	-	-	-	-	-	-
ATPase activity	13 / 98 4.35 E-06	-	-	-	-	-	-	-
nucleoside-triphosphatase activity	16 / 98 4.56 E-06	-	-	-	-	-	-	17 / 55 3.90 E-06
pyrophosphatase activity	16 / 98 7.59 E-06	-	-	-	-	-	-	17 / 55 8.20 E-06

**hydrolase activity, acting on acid anhydrides, in phosphorus-containing**	**16 / 98**<br>**7.90 E-06-**	**-**	**-**	**-**	**-**	**-**	**-**	**-**<br>**17 / 55**<br>**8.62 E-06**
**hydrolase activity, acting on acid anhydrides**	**16 / 98**<br>**8.06E-06**	**-**	**-**	**-**	**-**	**-**	**-**	**17 / 55**<br>**9.21 E-06**
**acetolactate synthase activity**	**3 / 98**<br>**8.30 E-06**	**-**	**4 / 63**<br>**2.19 E-08**	**-**	**4 / 54**<br>**9.41 E-09**	**-**	**-**	**-**
**glutamyl-tRNA reductase activity**	**3 / 98**<br>**8.30 E-06**	**-**	**-**	**-**	**-**	**-**	**-**	**-**
**glutamate synthase activity**	**3 / 98**<br>**8.30 E-06**	**-**	**-**	**-**	**-**	**-**	**-**	**-**
**biotin binding**	**-**	**-**	**3 / 63**<br>**7.00 E-06**	**-**	**-**	**-**	**-**	**-**
**metalloendopeptidase activity**	**-**	**-**	**-**	**-**	**-**	**-**	**-**	**8 / 55**<br>**1.80 E-08**
