# Supplementary material for: Protein disorder in plants: a view from the chloroplast
Source: BMC Plant Biol. 2012 Sep 13;12:165. doi: 10.1186/1471-2229-12-165 (PMC3460767; doi:10.1186/1471-2229-12-165)
Supplement: Additional file 6 — Table S5.Selection results for gene ontology categories in non-disordered proteins encoded by chloroplast genes and transferred to nuclear genome. A) biological process (P) GO categories; B) cellular components (C) GO categories; C) molecular function (F) GO categories. [file 1471-2229-12-165-S6.pdf]

Supplementary information

Table S3_A. Selection results for P-Branch gene ontology categories in non-disordered proteins encoded by chloroplast genes and transferred to nuclear genome.

GO annotation	AT	PT	VV	OS	SB	ZM	GM	PP	OT	MRC
**ribosome biogenesis**	**28 / 110 1.28 E-31***	-	-	-	-	-	-	-	-	-
**ribonucleo-protein complex biogénesis**	**28 / 110 3.44 E-31**	-	-	-	-	-	-	-	-	-
**response to external stimulus**	-	-	-	**20 / 62 2.0 E-13**	-	-	-	-	-	-
**metabolic process**	-	**118 / 157 1.91 E-15**	-	-	**54 / 62 1.58 E-11**	-	-	-	-	-
**translation**	**36 / 110 4.20 E-30**	**41 / 157 2.89 E-22**	**17 / 218 1.14 E-06**	**15 / 62 6.36 E-08**	**17 / 62 6.64 E-10**	**15 / 120 9.1 E-15**	**16 / 23 9.67 E-20**	**39 / 136 5.00 E-17**	-	**14 / 36 8.48 E-09**
**cellular ketone metabolic process**	-	-	-	**19 / 62 7.50 E-08**	-	-	-	-	-	-
**carboxylic acid metabolic process**	-	-	-	**18 / 62 5.26 E-07**	-	-	-	-	-	-
**oxoacid metabolic process**	-		-	**18 / 62 5.26 E-07**	-	-	-	-	-	-
**organic acid metabolic process**	-	-	-	**18 / 62 5.33 E-07**	-	-	-	-	-	-

cellular component biogenesis at cellular level	28 / 110<br>2.25 E-27	-	-	-	-	-	-	-	-	-
cellular component biogenesis	32 / 110<br>9.71 E-26	-	-	-	-	-	-	-	-	-
cellular macromolecule biosynthetic process	41 / 110<br>1.06 E-21	45 / 157<br>7.73 E-18	-	-	18 / 62<br>2.78 E-07	15 / 120<br>4.88 E-12	16 / 23<br>1.90 E-15	40 / 136<br>5.75 E-13	-	14 / 36<br>2.25 E-06
macromolecule biosynthetic process	41 / 110<br>1.91 E-21	45 / 157<br>9.17 E-18	-	-	18 / 62<br>2.87 E-07	15 / 120<br>5.11 E-12	16 / 23<br>2.08 E-15	40 / 136<br>6.37 E-13	-	14 / 36<br>2.00 E-06
gene expression	36 / 110<br>5.61 2E-21	42 / 157<br>7.04 E-17	-	-	17 / 62<br>4.07 E-07	15 / 120<br>3.02 E-12	16 / 23<br>2.16 E-16	40 / 136<br>8.95 E-13	-	15 / 36<br>1.22 E-06
cellular component organization or biogenesis at cellular level	35 / 110<br>1.38 E-20	-	-	-	-	-	-	-	-	-
cellular biosynthetic process	50 / 110<br>1.40 8E-20	76 / 157<br>2.53 E-30	35 / 218<br>3.04 E-09	-	24 / 62<br>1.23 E-08	17 / 120<br>6.56 E-12	17 / 23<br>5.70 E-13	76 / 136<br>1.35 E-25	15 / 26<br>8.5 5E-07	20 / 36<br>4.70 E-09

biosynthetic process	51 / 110<br>4.88 E-20	83 / 157<br>1.44 E-33	40 / 218<br>3.47 E-11	41 / 62<br>2.49 E-13	29 / 62<br>1.97 E-11	18 / 120<br>1.42 E-12	17 / 23<br>3.16 E-12	74 / 136<br>9.42 E-30	17 / 26<br>2.55 E-08	22 / 36<br>1.84 E-10
cellular component organization or biogenesis	36 / 110<br>1.85 E-17	-	-	-	-	-	-	-	-	-
metabolic process	76 / 110<br>3.35 E-17	-	70 / 218<br>5.22 E-08	54 / 62<br>1.75 E-09	-	22 / 120<br>1.35 E-06	-	103 / 136<br>1.02 E-14	-	-
cellular protein metabolic process	42 / 110<br>1.28 E-11	-	-	-	-	16 / 120<br>6.79 E-08	16 / 23<br>5.95 E-09	56 / 136<br>2.33 E-09	-	-
cellular metabolic process	59 / 110<br>5.61 E-11	91 / 157<br>2.08 E-09	54 / 218<br>7.93 E-06	46 / 62<br>2.52 E-08	-	19 / 120<br>9.68 E-07	-	86 / 136<br>8.79 E-14	-	-
cellular process	70 / 110<br>1.73 E-10	103 / -157<br>3.43 E-07	-	51 / 62<br>2.93 E-06	-	-	-	93 / 136<br>8.45 E-13	-	-

**cellular macromolecule metabolic process**	**47 / 110**<br>**3.02 E-10**	**-**	**-**	**-**	**-**	**17 / 120**<br>**3.73 E-07**	**16 / 23**<br>**5.6 E-07**	**40 / 136**<br>**5.75 E-13**	**-**	**-**
**protein metabolic process**	**43 / 110**<br>**4.56 E-10**	**-**	**-**	**-**	**-**	**17 / 120**<br>**1.19 E-07**	**16 / 23**<br>**8.05 E-08**	**61 / 136**<br>**1.92E-07**	**-**	**-**
**macromolecule metabolic process**	**48 / 110**<br>**6.74 E-09**	**-**	**-**	**-**	**-**	**17 / 120**<br>**5.43 E-06**	**16 / 23**<br>**4.56 E-06**	**-**	**-**	**-**
**lipid A biosynthetic process**	**5 / 110**<br>**7.00 E-09**	**-**	**-**	**-**	**-**	**-**	**-**	**-**	**-**	**-**
**lipid A metabolic process**	**5 / 110**<br>**7.00 E-09**	**-**	**-**	**17 / 62**<br>**1.05 E-06**	**-**	**-**	**-**	**-**	**-**	**-**
**lipopolysaccharide metabolic process**	**5 / 110**<br>**1.86 E-08**	**-**	**-**	**-**	**-**	**-**	**-**	**-**	**-**	**-**
**lipopolysaccharide biosynthetic process**	**5 / 110**<br>**1.86E-08**	**-**	**-**	**-**	**-**	**-**	**-**	**-**	**-**	**-**
**phylloquinone biosynthetic process**	**4 / 110**<br>**3.76 E-07**	**-**	**-**	**-**	**-**	**-**	**-**	**-**	**-**	**-**
**phylloquinone metabolic process**	**4 / 110**<br>**3.77 E-07**	**-**	**-**	**-**	**-**	**-**	**-**	**-**	**-**	**-**

**vitamin K biosynthetic process**	**4 / 110<br>1.12 E-06**	**-**	**-**	**-**	**-**	**-**	**-**	**-**	**-**	**-**
**vitamin K metabolic process**	**4 / 110<br>1.12 E-06**	**-**	**-**	**-**	**-**	**-**	**-**	**-**	**-**	**-**
**primary metabolic process**	**53 / 110<br>1.89 E-06**	**92 / 157<br>2.59 E-08**	**-**	**49 / 62<br>6.27 E-08**	**-**	**20 / 120<br>9.03 E-07**	**-**	**-**	**-**	**-**
**glycolipid metabolic process**	**5 / 110<br>1.991E-06**	**-**	**-**	**-**	**-**	**-**	**-**	**-**	**-**	**-**
**glycolipid biosynthetic process**	**5 / 110<br>1.99 1E-06**	**-**	**-**	**-**	**-**	**-**	**-**	**-**	**-**	**-**
**membrane lipid biosynthetic process**	**5 / 110<br>1.33 E-05**	**-**	**-**	**-**	**-**	**-**	**-**	**-**	**-**	**-**
**quinone cofactor metabolic process**	**4 / 110<br>9.93 E-05**	**-**	**-**	**-**	**-**	**-**	**-**	**-**	**-**	**-**
**quinone cofactor biosynthetic process**	**4 / 110<br>9.93 E-05**	**-**	**-**	**-**	**-**	**-**	**-**	**-**	**-**	**-**

**fat-soluble vitamin metabolic process**	**4 / 110 5.24 E-05**	**-**	**-**	**-**	**-**	**-**	**-**	**-**	**-**	**-**
**cofactor biosynthetic process**	**-**	**-**	**-**	**-**	**12 / 136 8.19 E-09**	**-**	**-**	**-**	**-**	**-**
**small molecule biosynthetic process**	**-**	**-**	**-**	**-**	**19 / 136 7.61 E-06**	**-**	**-**	**-**	**-**	**-**
**glutamate biosynthetic process**	**-**	**5 / 157 2.78 E-07**	**-**	**-**	**-**	**-**	**-**	**3 / 62 9.87 E-06**	**-**	**-**
**glutamine family amino acid biosynthetic**	-	**9 / 157 4.67 E-10**	-	-	-	-	-	-	-	-
**glutamine family amino acid metabolic process**	-	**9 / 157 4.57 E-09**	-	-	-	-	-	-	-	-
**organic acid biosynthetic process**	-	**18 / 157 7.58 E-08**	-	-	-	-	-	-	-	-
**carboxylic acid biosynthetic process**	-	**18 / 157 7.58 E-08**	-	-	-	-	-	-	-	-
**S-glycoside biosynthetic process**	-	**6 / 157 5.9485722 1623502E-06**	-	-	-	-	-	-	-	-

**glycosinolate biosynthetic process**	-	**6 / 157<br>5.95 E-06**	-	-	-	-	-	-	-	-
**glucosinolate biosynthetic process**	-	**6 / 157<br>5.95 E-06**	-	-	-	-	-	-	-	-
**glutamate metabolic process**	-	**5 / 157<br>6.42 E-06**	-	-	-	-	-	-	-	-

*** P-value cut-off = 1.0 E-5**

Table S3_B . Selection results for C-Branch gene ontology categories in non-disordered proteins encoded by chloroplast genes and transferred to nuclear genome.

GO annotation	AT	PT	VV	OS	SB	ZM	GM	PP	OT	MCR
**cytosolic large ribosomal subunit**	**32 / 110 1.05 E-46**	-	-	-	-	-	-	-	-	-
**peroxisome**	-	-	-	**12 / 62 8.22 E-17**	-	-	-	-	-	-
**cytosolic ribosome**	**40 / 110 2.54 E-46**	-	-	-	-	-	-	-	-	-
**ribosome**	**43 / 110 3.89E-45**	**38 / 157 1.87 E-24**	**16 / 218 1.44 E-08**	**15 / 62 2.07E-11**	**17 / 62 6.84 E-13**	**15 / 120 1.25 E-16**	**16 / 23 3.40 E-21**	**37 / 136 1.65 E-20**	-	**12 / 36 6.43 E-10**
**cytosolic part**	**40 / 110 5.97 E-45**	**73 / 157 3.69 E-18**	**47 / 218 4.88 E-14**	-	-	**20 / 120 9.88 E-06**	-	-	-	**13 / 36 1.06 E-06**
**large ribosomal subunit**	**32 / 110 1.04 E-43**	-	-	-	-	-	-	-	-	-
**ribonucleo protein complex**	**43 / 110 4.16 E-39**	**38 / 157 1.02 E-22**	**16 / 218 7.27 E-08**	**15 / 62 9.81 E-11**	**17 / 62 6.29 E-12**	**15 / 120 4.12E-16**	**16 / 23 1.47 E-20**	**38 / 136 6.63 E-20**	-	**12 / 36 8.59E-09**
**Cytosol**	**40 / 110 6.11 E-37**	-	-	**11 / 62 4.56 E-08**	-	-	-	-	-	-

**non-membrane-bounded organelle**	**43 / 110<br>9.28 E-30**	**38 / 157<br>1.81 E-14**	**-**	**-**	**17 / 62<br>6.42 E-09**	**15 / 120<br>1.28 E-12**	**16 / 23<br>1.10 E-16**	**37 / 136<br>7.49 E-14**	**-**	**12 / 36<br>1.25 E-06**
**intracellular non-membrane-bounded organelle**	**-**	**38 / 157<br>1.81 E-14**	**-**	**-**	**17 / 62<br>6.42 E-09**	**15 / 120<br>1.28 E-12**	**16 / 23<br>1.10 E-16**	**37 / 136<br>7.49 E-14**	**-**	**12 / 36<br>1.25 E-06**
**macromolecular complex**	**44 / 110<br>1.47 E-21**	**46 / 157<br>4.33 E-07**	**-**		**-**	**16 / 120<br>2.52 E-12**	**16 / 23<br>4.10 E-14**	**46 / 136<br>2.04 E-08**	**-**	-
**intracellular organelle part**	**50 / 110<br>1.98 E-21**	**-**	**-**	**-**	**-**	**-**	**-**	**-**	**-**	-
**organelle part**	**50 / 110<br>2.02 E-21**	**-**	**-**	**-**	**-**	**-**	**-**	**-**	**-**	-
**cytoplasmic part**	**71 / 110<br>1.51 E-20**	**-**	**-**	**-**	**-**	**-**	**16 / 23<br>1.73 E-07**	**40 / 136<br>1.39 E-13**	**-**	-
**cytoplasm**	**72 / 110<br>1.38 E-19**	**77 / 157<br>2.56 E-18**	**48 / 218<br>7.09 E-13**	**-**	**-**	**-**	**16 / 23<br>1.18 E-06**	**44 / 136<br>1.40 E-13**	**13 / 26<br>2.54 E-06**	**15 / 36<br>5.18 E-06**
**intracellular organelle**	**71 / 110<br>1.94 E-12**	**-**	**-**	**-**	**-**	**-**	**-**	**-**	**-**	-
**organelle**	**71 / 110<br>1.95 E-12**	**-**	**-**		**-**	**-**	**-**	**-**	**-**	-
**intracellular part**	**72 / 110<br>8.89 E-11**	**-**	**-**	**-**	**-**	**-**	**-**	**-**	**-**	-
**intracellular**	**72 / 110<br>1.24 E-09**	**-**	**-**	**-**	**-**	**-**	**-**	**-**	**-**	-

plastid	33 / 110<br>4.90 E-05	39 / 157<br>3.08 E-12	32 / 218<br>3.50 E-14	-	-	-	-	-	-	-	-
chloroplast	31 / 110<br>2.82 E-4	36 / 157<br>1.11 E-10	30 / 218<br>4.86 E-13	-	-	-	-	-	-	-	-
cytosolic small ribosomal subunit	6 / 110<br>6.00 E-4	6 / 157<br>1.30 E-06	-	-	-	-	-	-	-	-	-
microbody	-	-	-	12 / 62<br>8.22 E-17	-	-	-	-	-	-	-
small ribosomal subunit	-	9 / 157<br>3.90 E-07	-	-	-	-	-	-	-	-	-
plastid part	-	21 / 157<br>7.44 E-07	-	-	-	-	-	-	-	-	-
plastid stroma	-	15 / 157<br>7.68 E-07	-	-	-	-	-	-	-	-	-
chloroplast stroma	-	19 / 157<br>9.31 E-06	-	-	-	-	-	-	-	-	-

Table S3_C. Selection results for F-Branch gene ontology categories in non-disordered proteins encoded by chloroplast genes and transferred to nuclear genome.

GO annotation	AT	PT	VV	OS	SB	ZM	GM	PP	OT	MCR
**structural constituent of ribosome**	**41 / 110<br>4.59 E-45**	**36 / 157<br>1.08 E-22**	**14 / 218<br>5.27 E-07**	**15 / 62<br>1.55 E-11**	**17 / 62<br>1.02 E-12**	**-**	**-**	**37 / 136<br>1.09 E-19**	**-**	**-**
**structural molecule activity**	**41 / 110<br>3.87 E-39**	**36 / 157<br>2.05 E-19**	**-**	**15 / 62<br>2.72 E-06**	**17 / 62<br>2.68 E-11**	**-**	**-**	**37 / 136<br>2.04 E-17**	**-**	**-**
**UDP-3-O-[3-hydroxymyristoyl] acetylglucosamine deacetylase activity**	**5 / 110<br>1.32 E-10**	**-**	**-**	**-**	**-**	**-**	**-**	**-**	**-**	**-**
**AMP binding**	**5 / 110<br>2.76 E-09**	**4 / 157<br>1.28 E-06**	**5 / 218<br>5.55 E-09**	**-**	**-**	**-**	**-**	**-**	**-**	**-**
**deacetylase activity**	**5 / 110<br>2.54 E-06**	**-**	**-**	**-**	**-**	**-**	**-**	**-**	**-**	**-**
**hydrolase activity, acting on carbon -nitrogen (but not peptide) bonds, in l[illegible] amides**	**5 / 110<br>3.5 E-4**	**-**	**-**	**-**	**-**	**-**	**-**		**-**	**-**
**thiamine pyrophosphate binding**	**-**	**-**	**7 / 218<br>4.27 E-11**	**-**	**-**	**-**	**-**	**-**	**-**	**-**

acetolactate synthase activity	-	-	4 / 218<br>7.67 E-07	-	-	-	-	-	-	-
structural molecule activity	-	-	14 / 218<br>5.75 E-06	-	-	-	-	-	-	-
glutamate synthase activity	-	6 / 157<br>7.16 E-11	-	-	7 / 62<br>2.19 E-16	-	-	-	-	-
oxidoreductase activity, acting on the CH-NH2 group of donors	-	-	-	-	7 / 62<br>7.15 E-14	-	-	-	-	-
ligase activity, forming carbon-sulfur bonds	-	16 /157<br>3.35 E-18	-	-	-	-	-	-	-	-
4-coumaratE-CoA activity	-	12 / 157<br>1.29 E-15	-	-	-	-	-	-	-	-
CoA-ligase activity	-	13 / 157<br>3.81 E-15	-	-						
acid-thiol ligase activity	-	13 / 157<br>3.81 E-15	-	--	-	-	-	-	-	-
benzoate-CoA ligase activity	-	6 / 157<br>1.03 E-11	-	-	-	-	-	-	-	-

**two-component response regulator activity**	**-**	**11 / 157 1.66 E-08**	-	-	-	-	-	-	--	-
**acetylglutamate kinase activity**	-	**4 / 157 1.28 E-06**	-	-	-	-	-	-	-	-
**ligase activity**	-	**26 / 157 2.02 E-06**	-	-	-	-	-	-	-	-
**oxidoreductase activity, acting on the CH-NH2 group of donors**	-	**7 / 157 3.78 E-06**	-	-	-	-	-	-	-	-
**glutamate 5-kinase activity**	-	**4 / 157 8.91 E-06**	-	-	-	-	-	-	-	-
