# Supplementary material for: Protein disorder in plants: a view from the chloroplast
Source: BMC Plant Biol. 2012 Sep 13;12:165. doi: 10.1186/1471-2229-12-165 (PMC3460767; doi:10.1186/1471-2229-12-165)
Supplement: Additional file 7 — Table S6.Distribution of intrinsically disordered proteins in small (A) and large (B) ribosomal subunits from bacteria and plant chloroplast. [file 1471-2229-12-165-S7.pdf]

Table S4A: Distribution of intrinsically disordered proteins in small ribosomal subunit from bacteria and plant chloroplast

30S	Plants		PDB	Archae				Bacteria G+			Cyanobacteria				Eubacteria							Proteobacteria			
Protein name	Ath	Os	Tthe 2J00	Pyf	Meb	Mtj	Af	Myc	Bas	Myt	Nos	Pro	Syn	Sych	Bob	Chla	Chlb	Trep	Chlp	Clos	Aqa	Rip	Hep	Hai	Ec
S2			+	-	-	+	-	-	-	+	+	-	-	+	+	-	+	+	+	-	+	+	+	-	-
S3			+	-	+	-	+	+	-	+	+	+	+	-	+	+	+	+	-	-	-	-	-	+	-
S5	+	+	-	-	-	-	-	+	-	+	-	+	-	-	-	-	-	-	-	-	-	-	-	-	-
S6	+	+	-	-	ND	ND	ND	+	-	-	-	+	-	-	+	-	ND	-	-	-	-	-	+	+	+
S7			-	-	-	-	-	-	-	-	-	-	-	-	-	-	-	-	-	-	-	-	-	-	-
S8			-	-	-	-	-	-	-	-	-	-	-	-	-	-	-	-	-	-	-	-	-	-	-
S9/S4p	+	+	-	-	-	+	-	+	-	-	-	-	-	-	-	-	-	+	-	-	-	-	-	+	+
S10	+	+	-	-	-	-	-	-	-	-	-	-	-	-	-	-	-	-	-	-	-	-	-	-	-
S11	+	+	-	-	-	-	-	-	-	-	-	-	-	-	-	-	-	-	-	-	-	-	-	-	-
S12			-	-	ND	-	-	-	-	-	-	-	-	+	-	+	+	-	-	-	-	-	+	-	-
S13	+	+	-	-	-	-	-	-	-	-	-	-	-	-	-	-	-	-	-	-	-	-	-	-	-
S14			-	-	-	-	-	-	-	-	-	-	-	-	-	-	-	-	-	-	-	-	-	-	-
S15			-	-	-	-	-	-	-	-	-	-	-	-	-	-	-	-	-	-	-	-	-	-	-
S16/S9p			-	-	-	-	-	-	-	+	-	+	-	-	-	-	-	-	+	-	+	-	-	-	-
S17			-	-	-	-	-	-	-	-	-	-	-	-	-	-	-	-	-	-	-	-	-	-	-
S18			-	ND	ND	-	ND	+	-	-	-	-	-	-	+	-	-	+	-	-	-	+	-	-	-
S19			-	-	-	-	-	-	-	-	-	-	-	-	-	-	-	-	-	-	-	-	-	-	-
S20	+	+	-	ND	ND	ND	ND	-	-	-	-	-	-	-	ND	-	-	-	-	-	-	-	-	-	-
S21	+	+	-	ND	ND	ND	ND	-	-	ND	-	-	-	+	+	-	-	+	-	ND	-	+	-	+	+

[1]Ath, Arabidopsis thaliana; Os, Oryza sativa; Tthe, Thermus thermophlus; Pyf, Pyrococcus furiosus; Meb, Methanobacterium sp ; Mtj Methanocaldococcus jannaschii; Af, Archaeoglobus fulgidus; Myc, Mycoplasma pneumoniae; Bas, Bacillus subtilis; Myt, Mycobacterium tuberculosis; Nos, Nostoc punctiforme; Pro, Prochlorococcus marinus; Syn, Synechocystistis sp PCC 6803; Sych, Synechococcus sp; Bob, Borrelia burgdorferi; Chla, Chloroflexus aggregans; Chlb, Chlorobium chlorochromatii; Trep, Treponema pallidum; Chlp, Chlamydia pneumoniae; Clos, Clostridium hathewayi; Aqa, Aquifex aeolicus; Rip, Rickettsia prowazekii; Hep, Heliobacter pilori; Hai, Haemophilus influenzae; Ec, Escherichia coli.

[2]ND: protein not found.

Table S4B: Distribution of intrinsically disordered proteins in large ribosomal subunit from bacteria and plant chloroplast

50S	Plants		PDB	Archae				Bacteria G+			Cyanobacteria				Eubacteria							Proteobacteria			
Protein name	Ath	Os	Halo 1VQ8	Pyf	Meb	Mtj	Af	Myc	Bas	Myt	Nos	Pro	Syn	Sych	Bob	Chla	Chlb	Trep	Chlp	Clos	Aqa	Rip	Hep	Hai	Ec
**L1**	+	+	ND	-	-	-	-	-	-	-	-	-	-	-	-	-	-	-	-	-	-	-	-	-	-
**L2**	+	+	+	-	-	-	-	-	-	+	+	+	-	+	+	-	-	-	+	-	+	+	+	+	+
**L3**	+	+	-	-	-		-	+	-	+	-	+	+	+	+	+	+	-	-	-	-	+	+	-	+
**L4**	+	+	+	+	+	+	+	+	+	-	-	+	+	+	+	+	+	+	-	+	-	+	+	+	+
**L5**	+	+	+	-	-	-	-	-	-	-	-	-	-	+	-	-	-	-	-	-	-	-	-	-	-
**L6**	-	-	-	-	-	-	-	-	-	-	-	-	-	-	-	-	-	-	-	-	-	-	-	-	-
**L7/L12p**	+	+	ND	-	-	-	-	-	-	-	-	-	-	-	-	-	-	-	-	-	-	-	-	-	-
**L9**	+	+	ND	ND	ND	ND	ND	-	-	-	-	-	-	-	-	-	-	-	-	-	-	-	-	-	-
**L10**	-	-	+	-	-	-	-	-	-	-	+	-	-	-	-	-	-	-	-	ND	-	-	-	-	-
**L11**	+	+	-	-	-	-	-	-	-	-	-	-	-	-	-	-	-	-	-	-	-	-	-	-	-
**L13**	+	+	-	-	-	-	-	-	-	-	-	-	-	-	-	-	-	-	-	-	-	-	-	-	-
**L14**	-	-	-	-	-	-	-	-	-	-	-	-	-	-	-	-	-	-	-	-	-	-	-	-	-
**L15**	+	+	+	+	+	+	+	+	+	-	+	+	+	+	+	+	+	+	+	+	+	+	+	+	+
**L16**	-	-	ND	ND	ND	ND	ND	-	-	-	-	-	-	-	-	-	-	-	-	-	-	-	-	-	-
**L17**	+	+	-	ND	ND	ND	ND	-	-	-	-	-	-	-	-	-	-	-	-	-	-	-	-	-	-
**L18**	+	+	-	-	-	-	-	-	-	-	-	-	-	-	-	-	-	-	-	-	-	-	-	-	-
**L19**	+	+	-	-	-	-	-	-	-	-	-	+	-	-	-	-	ND	-	-	-	-	-	-	-	-
**L20**	-	-	ND	ND	ND	ND	ND	-	-	-	-	-	-	-	-	-	-	-	-	-	-	-	-	-	-
**L21**	+	+	-	-	-	-	-	-	-	-	+	-	-	+	-	-	-	-	-	-	-	-	-	-	-
**L22**	-	-	-	-	-	-	-	+	-	+	-	-	-	-	-	-	-	-	-	-	-	-	-	-	-
**L23**	-	-	-	-	-	-	-	+	-	-	-	-	-	-	-	-	-	-	-	-	-	-	-	-	-
**L24**	+	+	-	-	-	-	-	-	-	-	-	-	-	-	-	-	-	-	-	-	-	-	-	-	-
**L27**	-	-	ND	ND	ND	ND	ND	-	-	-	-	-	-	-	-	-	-	-	-	-	-	-	-	-	-
**L28**	+	+	ND	ND	ND	ND	ND	-	-	-	ND	-	-	-	-	-	-	-	-	-	-	-	-	-	-
**L29**	+	+	-	-	-	-	-	+	-	-	-	-	-	-	-	-	-	-	-	-	-	-	-	-	-
**L30**	-	-	-	-	-	-	-	-	-	+	ND	ND	ND	ND	-	-	-	-	ND	-	-	-	ND	-	-
**L31**	+	+	-	-	-	-	-	+	-	-	-	-	-	-	-	-	-	-	+	-	-	-	-	-	-
**L32**	-	-	-	-	-	-	-	-	-	-	-	-	-	-	ND	-	-	-	-	-	-	-	-	-	-
**L33**	-	-	ND	ND	ND	ND	ND	-	-	-	-	-	-	-	-	-	-	-	-	ND	-	-	-	-	-

**L34**	+	+	ND	-	-	-	ND	-	-	-	-	-	-	-	-	-	ND	-	-	-	-	-	ND	-	-
**L35**	+	+	ND	-	ND	ND	ND	-	-	-	-	-	-	-	-	ND	-	-	-	-	-	-	-	-	-
**L36**	+	+	ND	ND	ND	ND	ND	-	-	-	-	ND	-	-	-	-	-	-	-	ND	-	-	ND	-	-

[1]Ath, Arabidopsis thaliana; Os, Oryza sativa; Halo, Haloarcula marismortui; Pyf, Pyrococcus furiosus; Meb, Methanobacterium sp ; Mtj Methanocaldococcus jannaschii; Af, Archaeoglobus fulgidus; Myc, Mycoplasma pneumoniae; Bas, Bacillus subtilis; Myt, Mycobacterium tuberculosis; Nos, Nostoc punctiforme; Pro, Prochlorococcus marinus; Synechocystis sp PCC 6803; Sych, Synechococcus sp; Bob, Borrelia burgdorferi; Chla, Chloroflexus aggregans; Chlb, Chlorobium chlorochromatii; Trep, Treponema pallidum; Chlp, Chlamydia pneumoniae; Clos, Clostridium hathewayi; Aqa, Aquifex aeolicus; Rip, Rickettsia prowazekii; Hep, Heliobacter pylori; Hai, Haemophilus influenzae; Ec, Escherichia coli.

[2]ND: protein not found.
