# Supplementary material for: Protein disorder in plants: a view from the chloroplast
Source: BMC Plant Biol. 2012 Sep 13;12:165. doi: 10.1186/1471-2229-12-165 (PMC3460767; doi:10.1186/1471-2229-12-165)
Supplement: Additional file 8 — Table S7.Protein length of ribosomal proteins from bacteria and plant chloroplasts. [file 1471-2229-12-165-S8.pdf]

Table S5. Protein length of ribosomal proteins from bacteria and plant chloroplasts.

protein name	mean protein length		length plants		length bacteria																				
	plant	bacteria	Ath	Os	Pyf	Meb	Mtj	Af	Myc	Bas	Myt	Nos	Pro	Syn	Sych	Bob	Chla	Chlb	Trep	Chlp	Clos	Aqa	Rip	Hep	Hai	Ec
S2	237	254,4	237	237,237	202	198	222	197	294	246	287	264	222	269	267	260	238	251	291	277	269	274	296	292	240	241
S3	236,3	240,8	219	240,240, 240	198,210	299,205	208,222	229,211	273	218	274	260	244	240	248	293	241	253	247	223	218	212	217	234	235	233
S5	315,5	186,2	304	327	236	215	217	198	219	166	220	175	208	173	178	165	179	172	172	165	169	208	175	153	166	167
S6	214	122,9	208	220	ND	ND	ND	ND	215	95	96	116	138	113	96	139	137	ND	93	112	95	110	121	158	125	131
S7	156,5	164,8	156,156	157,157	215	187	191	194	155	156	156	156	156	156	156	157	157	155	156	157	156	160	160	155	156	179
S8	136	132	135	137	127,130	126,130	129,130	125,131	142	132	132	133	133	133	134	132	133	131	132	133	149	ND	132	131	130	130
S9/S4p	216,5	206,1	209	224	180,243	178,241	187,244	172,235	205	200	201	202	202	202	203	208	206	203	204	209	197	211	205	208	206	206
S10	189	107,4	192	186	102	102	106	106	108	102	101	105	106	105	105	103	102	103	102	105	181	104	105	104	103	103
S11	141,5	130,3	139	144	137	130	129	133	121	131	139	131	130	130	128	130	137	127	126	133	133	125	127	131	129	129
S12	126	132,8	126,126	126	147	ND	148	144	139	138	124	127	124	126	135	124	151	137	124	123	139	128	129	135	124	124
S13	171	127,8	170	172	148	149	150	146	124	121	124	126	121	127	127	125	126	125	121	122	122	126	125	120	118	118
S14	102,5	76,7	101	104	56	47	53	51	61	89,61	60,101	100	100	100	101	61	61	89	61	101	61	62	101	61	101	101
S15	89	101,2	89	ND	158	133	153	152	86	89	89	105	89	89	91	88	89	89	89	89	88	90	91	90	89	100
S16/S9p	71,5	135,2	80	63	135	133	136	135	132	130	151	138	135	137	137	116	132	129	129	145	130	147	161	129	130	130
S17	150	91,7	150	ND	67,113	106,63	117,63	65,111	85	87	135	82	88	81	83	84	89	89	84	86	67	107	77	86	85	84
S18	133	85,3	102	164	ND	ND	ND	ND	104	79	84,88	71	73	71	71	96,125	81	92	99	82	88	72	95	85	75	75
S19	93	111,5	93	ND	150,132	137,145	152,148	147,133	87	92	93	92	91	92	93	92	95	98	95	88	93	186	92	93	91	92
S20	200	95,8	203	197	ND	ND	ND	ND	87	88	86	110	98	97	100	ND	94	93	95	99	115	113	90	89	87	87
S21	183	65,2	218,184	147	ND	ND	ND	ND	71	57	ND	59,62	58	60	61	69	67	65	69	58	58	67	66	84	71	71
L1	353,5	230,5	347	360	216	212	232	215	226	232	235	237	235	238	236	226	237	229	226	232	231	242	238	234	229	234
L2	274,5	273,4	275,275	274,274	239	241	242	237	287	277	280	287	287	276	294	277	274	279	273	284	281	304	273	276	273	273
L3	272	239,7	272	272	365	337	335	331	287	209	217	221	218	213	214	206	210	209	208	219	210	241	216	191	208	209
L4	303,5	224	283	324	255	260	252	252	212	207	223	212	212	210	326	209	222	208	216	224	206	199	207	215	200	201
L5	271,5	183,5	263	280	186	168	190	178	180	190	187	182	179	211	180	182	180	195	185	180	179	188	179	181	179	179
L6	226	181,5	224	227,227	184	178	182	196	184	179	179	182	179	179	181	180	187	179	179	183	180	183	177	178	188	177
L7/L12	188	124,4	192,194,188	180,186	124	123	117	119	122	123,82	130	130	131	128	137	124	132	124	129	129	124	128	136	125	124	121
L9	193	154,2	198	188	ND	ND	ND	ND	149	149	152	152	152	152	153	173	153	151	156	169	148	149	171	149	149	149
L10	221	174,2	221	221	181	160	174	174	161	166	178	204	175	173	187	162	181	172	180	170	ND	199	169	164	163	165
L11	217	145,7	223	211	164	162	172	157	137	141	142	141	141	141	141	143	141	141	146	141	141	145	145	141	142	142
L13	238	145,5	242	234	142	140	137	156	146	145	147	153	150	151	151	133	143	149	142	149	144	144	155	141	142	142
L14	138,3	118,8	123	124,168	141,83	132,76	132,80	132	122	122	122	122	121	122	122	122	122	122	122	122	122	121	122	122	123	123
L15	271	157,1	278	264	194,147	145,181	143,194	194,179	151	146	146	147	150	147	157	145	170	184	153	144	146	149	150	135	144	144
L16	136,5	142,3	136	137	ND	ND	ND	ND	139	144	138	141	161	139	158	138	151	139	139	138	145	142	136	141	136	136
L17	210,5	134,9	211	210	155	153	ND	ND	124	120	180	116	116	116	116	123	116	154	164	142	178	118	136	116	128	127
L18	172,7	130,4	171	171,176	120,203	193,120	195,121	92,187	116	120	122	120	122	120	122	119	120	119	120	123	122	124	118	118	117	117
L19	226	127,7	230,226,223, 226,230	212,235	150	148	151	149	119	115	113	120	162	122	127	121	116	120	123	121	115	154	138	118	116	115
L20	119	118,8	118	120	ND	ND	ND	ND	127	119	129	118	115	117	118	115	119	115	122	121	118	118	117	116	117	118
L21	239,5	107,1	221,271	251,215	97	97	98	97	100	102	104	135	139	124	135	103	103	96	105	106	102	98	105	104	103	103
L22	140	131,2	161	150,109	155	153	156	155	184	113	208	118	126	121	123	120	113	119	124	111	128	98	119	122	110	110
L23	94	103,3	94,94	94,94	88	85	86	83	237	95	100	104	103	101	99	98	94	103	94	111	99	103	98	93	99	100
L24	195	103,1	199	191	66,124	117,53	120,70	58,120	111	103	105	117	118	115	132	101	110	89	114	111	101	132	113	73	103	104
L27	197,5	89,7	199	196	ND	ND	ND	ND	104	94	86	98	90	87	89	81	91	85	87	84	96	98	86	88	85	85
L28	141	76,5	144	138	ND	ND	ND	ND	65	62	94,78	ND	78	78	78	92	83,61	72	78	89	61	69	97	62	78	78

L29	170	72,1	174	166	83	68	70	68	111	66	77	75	70	73	78	66	68	65	72	72	68	73	71	66	63	63
L30	114	92,9	110	117,115	155,99	159,98	154,110	152,86	65	59	65	ND	ND	ND	ND	101	65	63	61	ND	60	125	63	ND	59	59
L31	140,5	80,3	145	136	98	81	87	88	97	66,82	80	80	86	81	73	81	99	70	67	109	69	68	78	67	70	70
L32	58,5	72,3	53	64	130	114	146	129	57	59	57	57	59	57	60	ND	61	62	62	60	59	63	65	48	56	57
L33	67	56,3	67	67	ND	ND	ND	ND	48,53	49,49	54,55	64	65	65	64	59	54	60	56	52	ND	61	56	52	56	55
L34	163	53,2	158	168	89	88	89	ND	48	44	47	44	45	45	46	51	57	51	51	45	44	47	44	ND	44	46
L35	146,5	67,5	146	147	87	ND	ND	ND	59	66	64	65	65	67	66	66	ND	64	66	64	65	67	67	64	89	65
L36	101,5	38,9	104	99	ND	ND	ND	ND	37	37	37	37	ND	38	38	37	38	38	38,37	45	ND	49	41	ND	37	38
